# TLR4 in POMC neurons regulates thermogenesis in a sex-dependent manner

**DOI:** 10.1016/j.jlr.2023.100368

**Published:** 2023-04-06

**Authors:** Yongxiang Li, Shuqing Zhu, Dan Du, Qiyong Li, Kailai Xie, Lvshuang Chen, Xiajie Feng, Xin Wu, Zhonghua Sun, Jingjing Zhou, Jinping Yang, Gang Shu, Songbo Wang, Ping Gao, Canjun Zhu, Qingyan Jiang, Lina Wang

**Affiliations:** 1 Guangdong Provincial Key Laboratory of Animal Nutrition Control, South China Agricultural University, Guangzhou, Guangdong, China; 2 National Engineering Research Center for Breeding Swine Industry, College of Animal Science, South China Agricultural University, Guangzhou, Guangdong, China; 3 Department of Genetics and Endocrinology, Guangzhou Women and Children's Medical Center, Guangzhou Medical University, Guangdong Provincial Clinical Research Center for Child Health, Guangzhou, China

**Keywords:** lipid balance, POMC, thermogenesis, TLR4

## Abstract

The rising prevalence of obesity has become a worldwide health concern. Obesity usually occurs when there is an imbalance between energy intake and energy expenditure. However, energy expenditure consists of several components, including metabolism, physical activity, and thermogenesis. Toll-like receptor 4 (TLR4) is a transmembrane pattern recognition receptor, and it is abundantly expressed in the brain. Here, we showed that pro-opiomelanocortin (POMC)-specific deficiency of TLR4 directly modulates brown adipose tissue thermogenesis and lipid homeostasis in a sex-dependent manner. Deleting TLR4 in POMC neurons is sufficient to increase energy expenditure and thermogenesis resulting in reduced body weight in male mice. POMC neuron is a subpopulation of tyrosine hydroxylase neurons and projects into brown adipose tissue, which regulates the activity of sympathetic nervous system and contributes to thermogenesis in POMC-TLR4-KO male mice. By contrast, deleting TLR4 in POMC neurons decreases energy expenditure and increases body weight in female mice, which affects lipolysis of white adipose tissue (WAT). Mechanistically, TLR4 KO decreases the expression of the adipose triglyceride lipase and lipolytic enzyme hormone-sensitive lipase in WAT in female mice. Furthermore, the function of immune-related signaling pathway in WAT is inhibited because of obesity, which exacerbates the development of obesity reversely. Together, these results demonstrate that TLR4 in POMC neurons regulates thermogenesis and lipid balance in a sex-dependent manner.

## GRAPHICAL ABSTRACT

**Figure:**
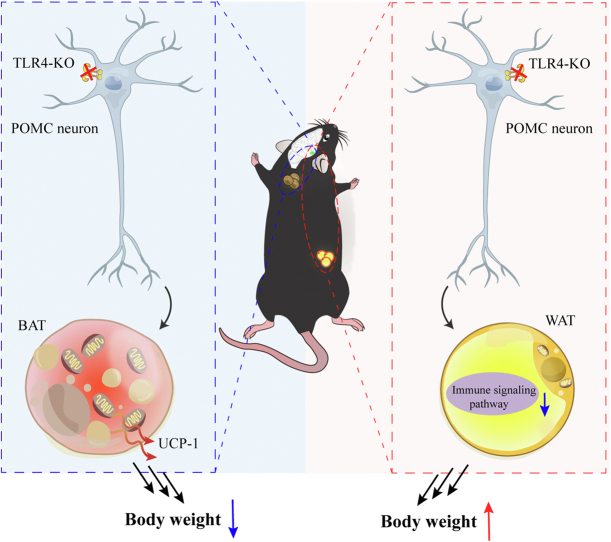

There is a delicate balance between energy intake and expenditure, which is strictly regulated by the homeostasis mechanism to maintain normal energy balance. Obesity usually occurs when there is a significant imbalance between energy intake and energy expenditure (1). Long-term increased food intake and decreased energy expenditure lead to the storage of energy, resulting in body weight gain and obesity (2, 3). However, energy expenditure consists of several components, including metabolism, physical activity, and thermogenesis. It is reported that reduced adaptive thermogenesis is the cause of reduced energy consumption during energy deficiency (4). In several brain regions involved in peripheral interaction, the hypothalamus plays an important role in coordinating the regulation of energy homeostasis (5).

The hypothalamus plays an important role in the regulation of energy homeostasis controlling feeding behavior and energy metabolism in mammals (6). Arcuate nucleus of the hypothalamus (ARH) is located in the base of the hypothalamus, adjacent to the median hump that has a semipermeable blood-brain barrier (7). The key to this process is two sets of interconnected ARH neurons. One set is the anorexigenic neurons expressing pro-opiomelanocortin (POMC) and cocaine- and amphetamine-regulated transcript (reduce food intake and increase metabolic processes), and the orexigenic neurons expressing neuropeptide Y (NPY) and agouti-related peptide (AGRP) (increase food intake and decrease metabolic processes), which have been shown to be involved in the control of metabolism in peripheral tissues (8, 9).

POMC neurons are essential in the regulation of negative energy balance. The anorexia of POMC neurons was discovered in POMC KO mice, which are overly hyperphagic and obese (10). When POMC neurons were activated, melanocyte-stimulating hormone will be released and binds to the melanocortin receptor 3/4, leading to reduced food intake and increased energy expenditure (11). Optogenetics showed that the direct activation of POMC neurons resulted in reducing food intake (12, 13). As expected, genetic mutations in the *Pomc* gene or disruption of the MC4R reduces energy expenditure and leads to obesity in rodents and humans (10, 14, 15). This provides further important evidence for POMC neuron in the physiological control of energy expenditure.

In addition to regulating appetite, POMC neurons also indirectly affect adipose tissue function and lipid metabolism (16, 17). Previous research has reported that partial CR6-interacting factor 1 deficiency in POMC neurons increased mitochondrial stress and activated thermogenesis in the adipose tissues (18). Serum- and glucocorticoid-regulated kinase 1 deletion in POMC neurons also exhibited increased adiposity and decreased energy expenditure (19). Besides, specific deletion of inositol-requiring enzyme 1α in POMC neuron impaired thermogenic responses in both brown adipose tissue (BAT) and inguinal white adipose tissue (iWAT) in response to cold environment (20). Losing autophagy-related 7 (*Atg7*) in POMC neuron leads to the failure of lipid utilization in BAT during fasting and cold exposure (21). In addition, the loss of POMC neuron-specific tyrosine phosphatase proteins reduced fat mass in iWAT and epididymal white adipose tissue (eWAT), and increased BAT thermogenesis, whereas food intake and overall body weight did not change (22).

The regulation of thermogenesis of BAT is under tight control by the central nervous system (CNS) (23), and in particular, the ARH has attracted special attention. BAT has become a possible target for normal control and treatment of adult obesity by regulating cold-induced and adaptive thermogenesis (24). There is a key hub linking between the CNS to peripheral organs through the sympathetic nervous system (SNS). The main function of the SNS in adipose tissue is to regulate energy balance by controlling thermogenesis, lipolysis, and fat mobilization (25, 26). The activation of SNS promotes the release of norepinephrine (NE) that binds to β-adrenergic receptor 3 (β3-AR), stimulating BAT thermogenesis and WAT lipolysis (27, 28, 29, 30). Because of sympathetic innervation, the release of NE in BAT leads to an increase in the expression of uncoupling protein 1 (UCP1), the mitochondrial proton carrier in BAT, which synthesizes the respiratory chain with ATP and generate heat (31, 32). The further mechanism of these phenotypes is the regulation of sympathetic nerve outflow from the CNS to peripheral tissues through affecting POMC neuronal activity (33). POMC neurons in the ARH have been shown to project to BAT across presynapses (34).

The Toll-like receptor 4 (TLR4) is a member of the TLR family, a kind of pattern recognition receptor and has a prominent role in activation of innate immune responses (35). TLR4 is known to cause obesity-associated insulin resistance (36, 37). In ARH, TLR4 is mainly expressed in microglia (38). Recent studies have shown that TLR4 can also be expressed in ventral tegmental area (VTA) dopamine neurons (39). TLR4-dependent hypothalamic inflammation plays a key role in the induction of metabolic disorders. The activation of TLR4 signal in hypothalamus can trigger hypothalamic inflammatory response and result in resistance to anorexia signal, which plays a key role in the occurrence of obesity (40). Another study showed that exposing to lipopolysaccharide (LPS) activates TLR4 to affect the activity of ARH neurons and feeding behavior. The inhibition of hypothalamic TLR4 by i.c.v. injection of anti-TLR4 antibody improves liver insulin signal transduction and reduces steatosis and gluconeogenesis (41). Loss or inhibition of TLR4 or myeloid differentiation primary response 88 in the hypothalamus restores energy homeostasis in rodents exposing high-fat diet (HFD) (37, 42, 43). Inhibition of TLR4-induced inflammation has been reported to reduce fatty acid-induced hypothalamic inflammation and hypothalamic injury (44). Knockdown of TLR4 in ARH protects obese rats from diet-induced weight gain, and energy intake has been demonstrated (35). Recent evidence suggests that inflammation of the hypothalamus affects the number of POMC and AGRP neurons (45, 46) and interferes the response to glucose, insulin and leptin, which can lead to obesity caused by HFD (47).

Activation of TLR4 by fatty acids, especially palmitic acid, is a key mediator between the HFD-induced inflammatory response and obesity and insulin resistance (37). Gene deletion of TLR4 can reverse HFD-mediated body weight gain and improve metabolic dysfunction (48). TLR4 KO also can resist endoplasmic reticulum stress induced by HFD in mice (49). These studies suggest that hypothalamic TLR4 may be an attractive target for the treatment of diet-induced obesity. Although TLR4 plays an important role in obesity and metabolic dysfunction, it remains unclear about the specific role of TLR4 in POMC neurons. In our current study, we investigated the specific role of TLR4 in hypothalamic POMC neurons by depleting TLR4 using virus strategy. Our results showed that TLR4 in POMC neurons exhibit a dimorphic response to thermogenesis and lipid homeostasis in a sex-dependent manner. Also, the underlined mechanism of these effects was further studied.

## MATERIALS AND METHODS

### Animals

POMC-enhanced GFP mice are the gift from Zhujiang Hospital, Southern Medical University, Guangzhou, China. TLR4^flox/flox^ (TLR4^f/f^, stock no.: 024872) mice were purchased from the Jackson Laboratory (Bar Harbor, ME). Male POMC-GFP-TLR4^f/+^ and female TLR4^f/+^ mice were generated by crossing male POMC-GFP mice and female TLR4^f/f^. To generate POMC-GFP-TLR4^f/f^ mice (both male and female), male POMC-GFP-TLR4^f/+^ mice were interbred with female TLR4^f/+^mice. The TLR4^f/f^ littermates were used as controls (both male and female). Mice were housed under conditions of controlled temperature (24°C) and illumination (12 h light/12 h dark cycle) with ad libitum access to food and water. All procedures performed on the mice followed the guidelines for animal care and were approved by the Animal Care and Use Committee of South China Agricultural University.

### Stereotaxic microinjection of virus vectors

In order to delete TLR4 in POMC neurons, AAV1-EF1a-N-CretrcintG (N-Cre, 1.1 × 10^13^ GC/ml; Addgene, catalog no.: 69570) and AAV1-EF1a-C-CreintG (C-Cre, 1.5 × 10^13^ GC/ml, Addgene, catalog no.: 69571) were used, which can express Cre in selectively GFP-labeled neurons (50). For adeno-associated virus (AAV) infection of the ARH POMC neurons, 8 week control, and POMC-GFP-TLR4^f/f^ mice, either sex was anesthetized with 4–5% isoflurane and maintained under 1–1.5% isoflurane throughout the surgery by using stereotaxic instrument (RWD Life Science). A total volume of 600 nl of the following viral constructs: 1:1:1 mix of AAV1-EF1a-N-CretrcintG, AAV1-EF1a-C-CreintG, and AAV1-FLEX-tdTomato (1.6 × 10^13^ GC/ml, Addgene, catalog no.: 28306) were injected into ARH in four injection sites (anterior-posterior: −1.46 mm and −1.7 mm, medial-lateral: ±0.35 mm, and dorsal-ventral: −5.8 mm). The pipette remained for 10 min at the end of infusion to allow virus diffusion. To determine whether POMC neurons project to BAT and iWAT directly, 8-week-old POMC-GFP male mice were dissected, and interscapular BAT and iWAT were exposed under 1–1.5% isoflurane. For retrograde synaptic tracers from BAT and iWAT, pAAV-CMV-WGA-NLS-Cre-P2A-EBFP2-WPRE (1.78 × 10^13^ GC/ml; Obio Technology, Shanghai; catalog no. H17837) was used (50). Six 50 nl nanoinjection virus was injected bilaterally into the BAT and iWAT, respectively. Meanwhile, 400 nl AAV1-FLEX-tdTomato virus was bilaterally injected into the ARH (anterior-posterior: −1.46 and −1.7 mm, medial-lateral: ±0.35 mm, and dorsal-ventral: −5.8 mm) of these mice. At 4 weeks postviral infections, mice were then perfused with 0.9% saline and 4% paraformaldehyde. Brains were immediately postfixed in ice-cold 4% paraformaldehyde overnight, after which they were cryoprotected in 30% sucrose in PBS. The brain sections (28 μm) were visualized via a Nikon imaging system (Nikon). The numbers of neurons (tdTomato- and POMC-GFP-positive) were counted by ImageJ (Rawak Software, Inc, Stuttgart, Germany).

### Body temperature, thermal imaging, and cold exposure

The rectal body temperature of control and POMC-TLR4-KO mice at 13 weeks after surgery of both males and femalse was measured in a room temperature environment at 9 AM and 9 PM by Thermalert TH-5 equipment (Physitemp Instruments, Inc, NJ), respectively. Interscapular temperature was assessed and visualized using a high-resolution infrared camera (E60bx: Compact-Infrared-Thermal Imaging-Camera; FLIR) and analyzed with a FLIR-Tools specific software package (51). For cold exposure experiments, mice were transferred from room temperature to 4°C, with free access to food and water. Then body temperature and interscapular temperature were recorded.

### RNA-sequencing analysis

iWAT samples used in RNA sequencing (RNA-Seq) were selected from control (*n* = 3) and POMC-TLR4-KO (*n* = 3) female mice. RNA extraction was performed by Hipure Universal RNA Kit (Magen Biotech Co, Ltd, Guangzhou, China). The primers of each gene are listed in Table 1. The RNA-Seq was analyzed by HiSeq® (Novogene Corp, China). Transcript with a q value ≤0.01 and Log2 fold change ≥1.5 was marked to be significantly different. The Database for Annotation, Visualization, and Integrated Discovery (https://magic.novogene.com) was applied to obtain differentially expressed genes (fold change ≥1.5) and to cluster genes.

**Table 1:** Primers sequences of genes used for real-time PCR

Target genes	Forward primer (5′-3′)	Reverse primer (5′-3′)
*TLR4*	ATG GCA TGG CTT ACA CCA CC	GAG GCC AAT TTT GTC TCC ACA
*Ppargc1a*	AAG TGT GGA ACT CTC TGG AAC TG	GGG TTA TCT TGG TTG GCT TTA TG
*PPARγ*	GGA AGA CCA CTC GCA TTC CTT	GTA ATC AGC AAC CAT TGG GTC A
*Prdm16*	CAG CAC GGT GAA GCC ATT C	CAG CAC GGT GAA GCC ATT C
*UCP1*	TTG GGC TTC TAT GCT GGG AG	GTG AAT GCT ATG CTC TTC TGT CT
*ATGL*	CTG AGA ATC ACC ATT CCC ACA TC	CAC AGC ATG TAA GGG GGA GA
*FASN*	GGA GGT GGT GAT AGC CGG TAT	TGG GTA ATC CAT AGA GCC CAG
*β3-AR*	ACT GCT AGC ATC GAG ACC TTG	AAG GGT TGG TGA CAG CTA GG
*β-actin*	GG CTG TAT TCC CCT CCA TCG	CCA GTT GGT AAC AAT GCC ATG T

### Statistical analysis

All data are presented as the means ± SEM. Statistical analysis was performed using GraphPad Prism 8.0 (GraphPad Software, Inc). Student’s *t*-test was used to determine significant differences between the control and POMC-TLR4-KO groups. Two-way ANOVA was used for multiple comparisons followed by post hoc Bonferroni test. A significance (alpha) level of *P* < 0.05 was considered statistically significant.

## RESULTS

### Generation and validation of mice lacking TLR4 in POMC neurons

**Fig. 1:**
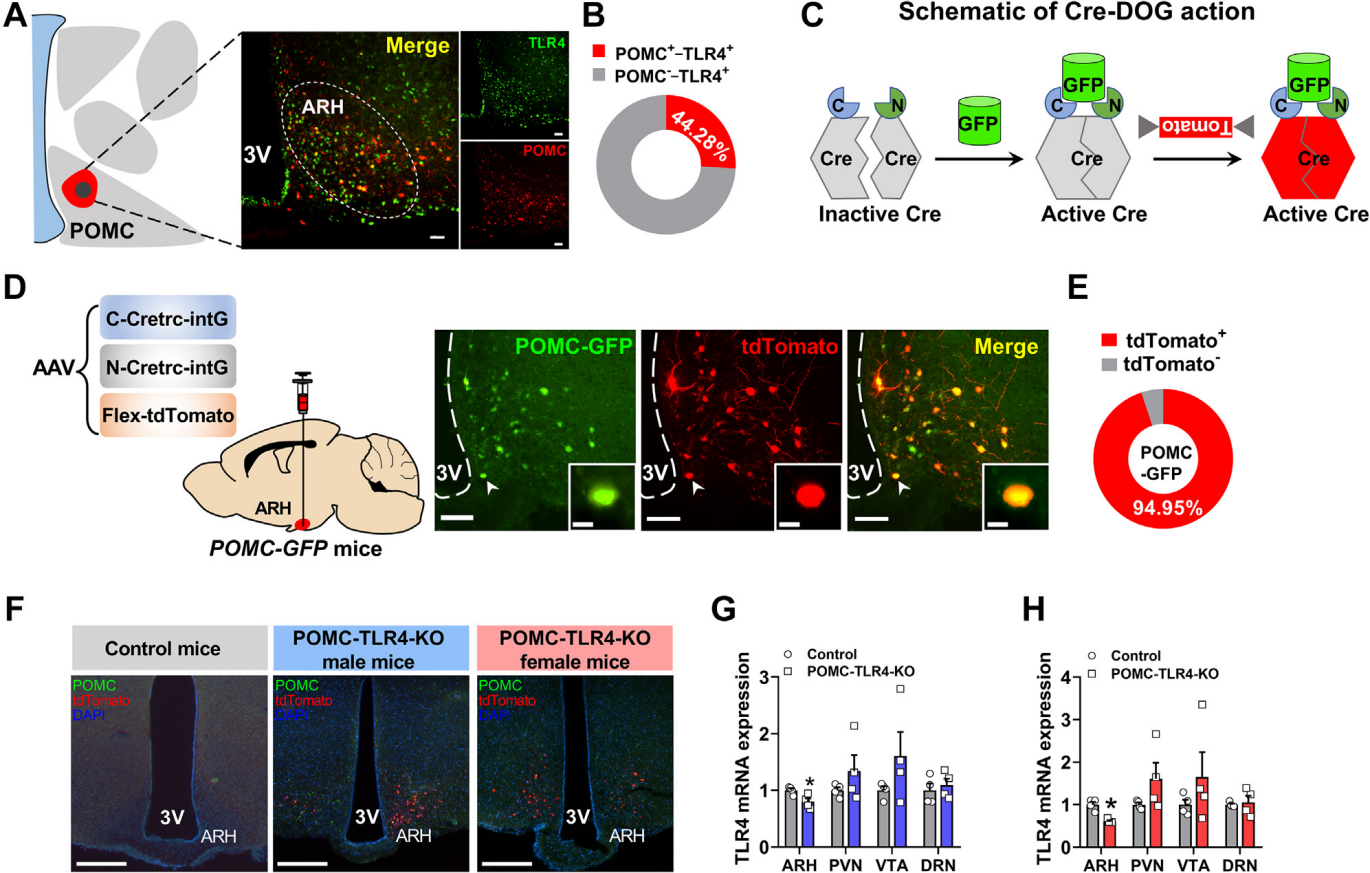
POMC neuron-specific deficiency of TLR4 in the ARH. A and B: The TLR4 expression in POMC neurons was measured by immunohistochemistry. Scale bars represent 200 μm. C: Schematic diagram of the CRE-DOG (GFP-dependent) system. The complementary cleavage components of Cre bind to GFP, inducing Cre recombinase-mediated activity. D and E: The rAAV mix (N-Cre, C-Cre, and Flex-tdTomato) was injected into ARH in POMC-GFP mice, and tdTomato reporter (red) is expressed only in GFP-positive neurons (green). Scale bars represent 200 and 40 μm. F: Male and female POMC-GFP mice show tdTomato fluorescence in the ARH indicating transduction and recombination of the AAV-(CRE-DOG); equivalently manipulated control mice (TLR4^f/f^ mice) do not. Scale bars represent 200 μm. G and H: *TLR4* mRNA expression in different tissues in both male and female mice. For male: ARH, control: *n* = 4, POMC-TLR4-KO: *n* = 6; PVN, VTA, and DRN, control: *n* = 4, POMC-TLR4-KO: *n* = 4. For female: ARH, PVN, VTA, and DRN, control: *n* = 4, POMC-TLR4-KO: *n* = 4. Data are expressed as the mean ± SEM. ∗*P* < 0.05, ∗∗*P* < 0.01. DRN, dorsal raphe nucleus.

TLR4 expresses in a variety of immune-related cell types, including microglia (52), astrocytes (53) and oligodendrocytes (54). In addition, TLR4 also can express in cortical neurons as well as neuronal progenitor (55, 56). However, whether TLR4 can express in POMC neurons and its role are unknown. To address this question, we first determined the expression of TLR4 in POMC neurons. Using immunofluorescence, TLR4 and POMC-GFP-positive neurons within ARH were identified, which accounts for about 26% of POMC neurons in ARH (Fig. 1A, B).

To further study the specific role of TLR4 in POMC neurons, we used the Cre recombinase dependent on GFP (CRE-DOG), a split component system that uses GFP and its derivatives to directly induce Cre/loxP recombination as described previously (50, 57)(Fig. 1C). Using AAV viral vectors, we delivered CRE-DOG to POMC-GFP mice, leading to effective recombination selectively in POMC-GFP labeled neurons after 4 weeks. Combination with the AAV-Flex-tdTomato, we successfully used GFP to control the activity of Cre (Fig. 1D). Meanwhile, colonization of tdTomato and POMC-GFP also showed that tdTomato accounts for about 95% of POMC neurons in ARH (Fig. 1E). Fluorescence immunohistochemistry showed the colocalization of tdTomato and POMC neurons in the ARH from bregma −2.18 mm to −1.46 mm (supplemental Fig. S2). This indicates successful targeting, transduction, and recombination of the CRE-DOG construct in our experiment.

To test the specific role of TLR4 in POMC neurons, first, we bilaterally delivered AAV mix (N-Cre, C-Cre, and Flex-tdTomato) into the ARH of TLR4^f/f^ and POMC-GFP-TLR4^f/f^ mice by stereotaxic injection. Both male and female mice show tdTomato fluorescent reporter expression in ARH successfully (Fig. 1F). *TLR4* mRNA expression was consistently decreased 50% in the ARH in both male and female mice but not in other brain areas (Fig. 1G, H). In addition, the loss of TLR4 in POMC neurons did not affect brain weight and size (supplemental Fig. S3). Taken together, these results confirm that TLR4 is successfully deleted in POMC neurons without affecting normal brain function.

### Selective deletion of TLR4 in POMC neurons regulates energy balance in a sex-dependent manner

**Fig. 2:**
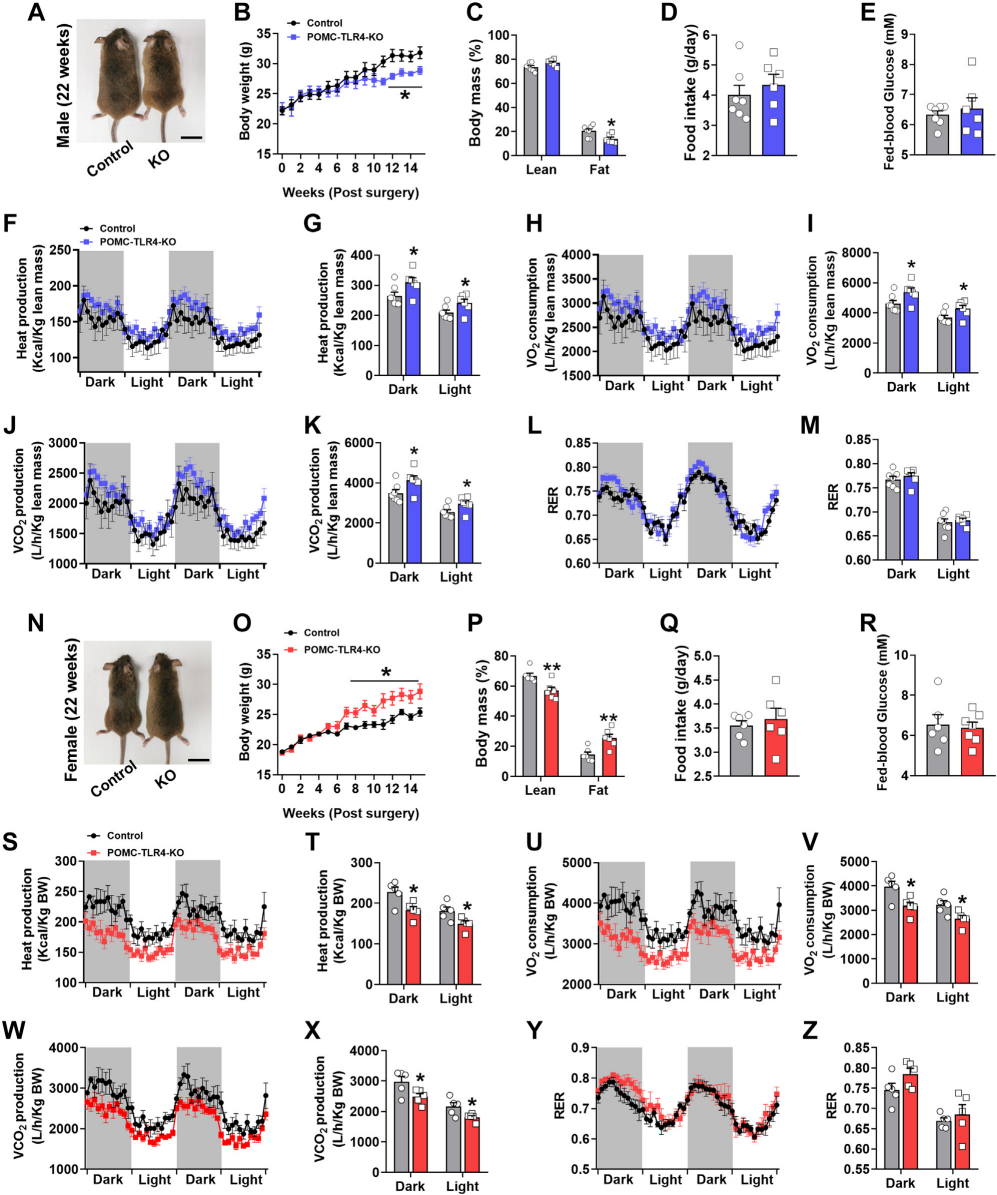
Specific deletion of TLR4 in POMC neurons is required for the regulation of energy balance in male and female mice. A–C: Representative body images, body weights, lean mass, and fat mass in control and POMC-TLR4-KO male mice at the indicated ages. For male: control: *n* = 7, POMC-TLR4-KO: *n* = 6. Scale bar represents 2 mm. D and E: Food intakes and fed blood glucose measured at the indicated ages. For male: control: *n* = 7, POMC-TLR4-KO: *n* = 6. F: Dynamic changes in heat production during dark and light phases. G: Heat production during dark and light phases. For male: control: *n* = 7, POMC-TLR4-KO: *n* = 6. H: Dynamic changes in O_2_ consumption during dark and light phases. I: An increase in O_2_ consumption was observed during the dark and light phases. For male: control: *n* = 7, POMC-TLR4-KO: *n* = 6. J: Dynamic changes in CO_2_ production during dark and light phases. K: An increase in CO_2_ production was observed during the dark and light phases. For male: control: *n* = 7, POMC-TLR4-KO: *n* = 6. L: Dynamic changes in respiratory exchange ratio during light and dark phases. M: No difference in RER was detected between the two groups of mice. For male: control: *n* = 7, POMC-TLR4-KO: *n* = 6. N–P: Representative body images, body weights, lean mass, and fat mass in control and POMC-TLR4-KO female mice at the indicated ages. For female: control: *n* = 6, POMC-TLR4-KO: *n* = 6. Scale bar represents 2 mm. Q and R: Food intakes and fed blood glucose measured at the indicated ages. For female: control: *n* = 6, POMC-TLR4-KO: *n* = 6. S: Dynamic changes in heat production during dark and light phases. T: Heat production during dark and light phases. For female: control: *n* = 5, POMC-TLR4-KO: *n* = 5. U: Dynamic changes in O_2_ consumption during dark and light phases. V: A decrease in O_2_ consumption was observed during the dark and light phases. For female: control: *n* = 5, POMC-TLR4-KO: *n* = 5. W: Dynamic changes in CO_2_ production during dark and light phases. X: A decrease in CO_2_ production was observed during the dark and light phases. For female: control: *n* = 5, POMC-TLR4-KO: *n* = 5. Y: Dynamic changes in RER during light and dark phases. Z: No difference in RER was detected between the two groups of mice. For female: control: *n* = 5, POMC-TLR4-KO: *n* = 5. Data are expressed as the mean ± SEM. ∗*P* < 0.05, ∗∗*P* < 0.01. RER, respiratory exchange ratio.

Next, we examined the effect of TLR4 depletion on energy balance regulation. Male POMC-TLR4-KO mice exhibited significantly an age-dependent decreased body weight from the 10 weeks after injecting the virus compared with control mice (Fig. 2A, B). Body composition analysis revealed that male POMC-TLR4-KO mice decreased fat mass but unchanged lean mass at 14 weeks after injection (Fig. 2C). In addition, no significant differences in food intake (Fig. 2D) and fed blood glucose were found between control and KO mice (Fig. 2E), but the heat production (Fig. 2F, G), O_2_ consumption (Fig. 2H, I), and CO_2_ production (Fig. 2J, K) were markedly increased, and the respiratory exchange ratio (Fig. 2L, M) was not changed in male POMC-TLR4-KO mice compared with control mice. However, female POMC-TLR4-KO mice displayed opposite phenotypes that observed in male mice. Female POMC-TLR4-KO mice significantly exhibited an age-dependent increased body weight from the 8 weeks after injecting the virus compared with control mice (Fig. 2N, O). Body composition analysis revealed that male POMC-TLR4-KO mice increased fat mass and decreased lean mass at 14 weeks after injection (Fig. 2P). Food intake and fed blood glucose were normal (Fig. 2Q, R), but the heat production (Fig. 2S, T), O_2_ consumption (Fig. 2U, V), and CO_2_ production (Fig. 2W, X) were markedly decreased, and the respiratory exchange ratio (Fig. 2Y, Z) was also not changed in female POMC-TLR4-KO mice. Again, the glucose homeostasis (supplemental Fig. S4A–F) and anxiety-like behaviors (elevated plus maze and open field test) (supplemental Fig. S5A–J) were not changed in both male and female POMC-TLR4-KO mice. However, in multiple feeding behavior, deletion of TLR4 only increase the number of meals in dark phase (supplemental Fig. S6A) in male mice without affecting other feeding behavior (food intake, time in feeding, average interintake interval), and physical activity (supplemental Fig. S6B–E). There is no difference in all multiple feeding and physical activities in POMC-TLR4-KO female mice (supplemental Fig. S6F–J). Together, these data suggest that TLR4 in POMC neurons is required for energy balance regulation in a sex-dependent manner.

### Genetic deletion of TLR4 in the POMC stimulated BAT thermogenesis only in male mice

**Fig. 3:**
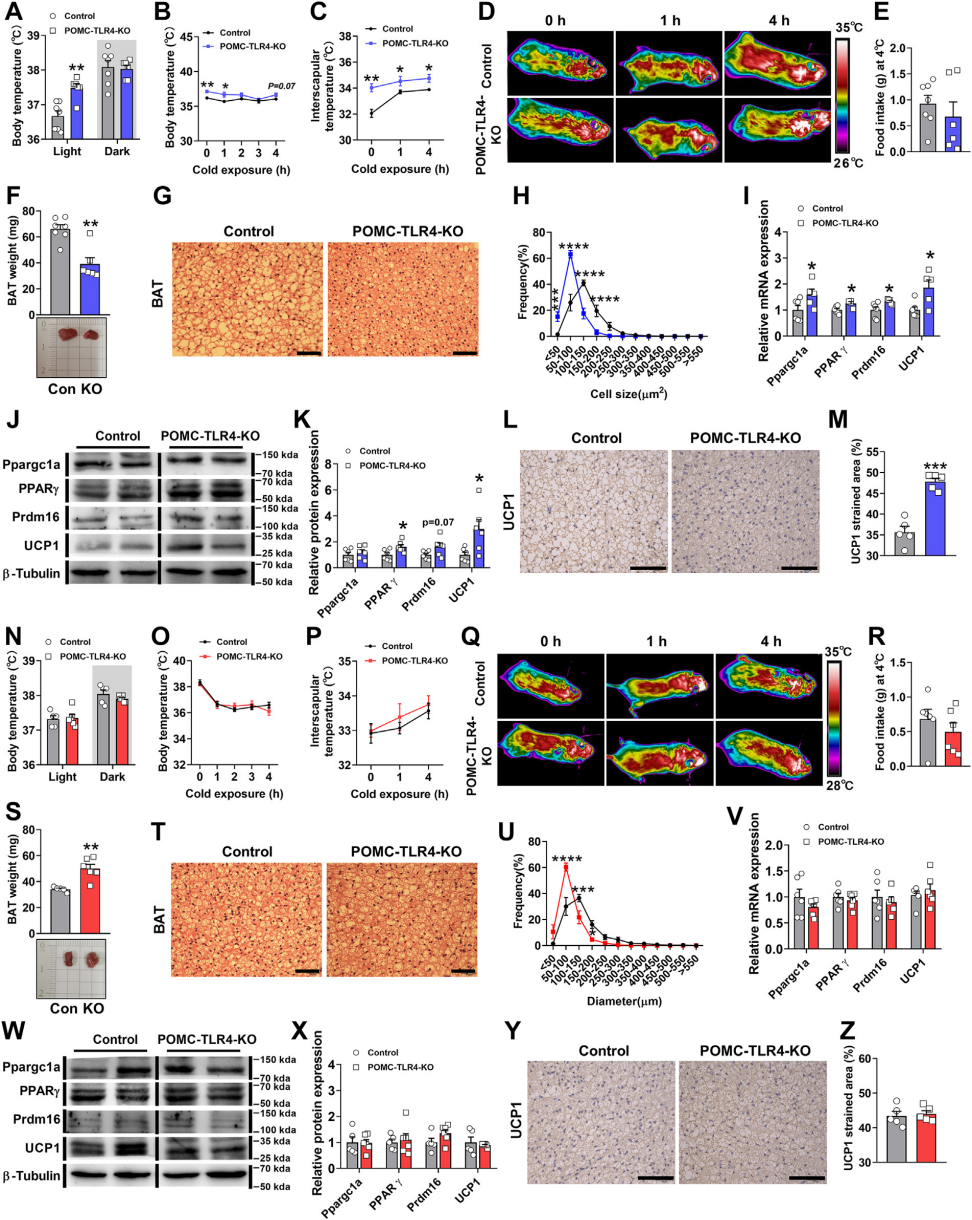
Loss of TLR4 in POMC neurons affects thermogenesis only in male mice but not female mice and lipid mobilization in male and female mice. A: Rectal temperature of male mice measured at room temperature environment at 12 weeks after injection. For male: control: *n* = 7, POMC-TLR4-KO: *n* = 6. B: Rectal temperature of male mice measured at cold environment at 12 weeks after injection. For male: control: *n* = 7, POMC-TLR4-KO: *n* = 6. C: BAT temperature of male mice measurements during room temperature environment and cold challenge at 12 weeks after injection virus. For male: control: *n* = 7, POMC-TLR4-KO: *n* = 6. D: Representative near-infrared analysis of male mice. E: Quantification of food intake of male mice after cold exposure. For male: control: *n* = 7, POMC-TLR4-KO: *n* = 6. F: Weight and representative photographs of BAT at 14 weeks after injection in male mice. For male: control: *n* = 7, POMC-TLR4-KO: *n* = 6. G and H: Representative H&E-stained sections and frequency distribution of adipocyte cell size of BAT and iWAT in male mice. Scale bar represents 40 μm. I: Changes in the mRNA expression levels involved in the BAT thermogenesis between two groups of mice. For male: *Ppargc1a*, *Prdm16*, and *Ucp1*, control: *n* = 6, POMC-TLR4-KO: *n* = 5. *Pparγ*, control: *n* = 6, POMC-TLR4-KO: *n* = 4. J: Western blot analysis of Ppargc1a, PPARγ, Prdm16, UCP1, and β-tubulin content in BAT of controls and POMC-TLR4-KO male mice. K: Quantification of Western blot analysis in BAT samples from controls and POMC-TLR4-KO male mice. For male: control: *n* = 6, POMC-TLR4-KO: *n* = 5. L and M: Immunostaining of UCP1 (L) and quantification (M) in BAT. Scale bar represents 20 μm. For male: control: *n* = 5, POMC-TLR4-KO: *n* = 5. N: Rectal temperature of female mice measured at room temperature environment at 12 weeks after injection. For female: control: *n* = 5, POMC-TLR4-KO: *n* = 6. O: Rectal temperature of female mice measured at cold environment at 12 weeks after injection. P: BAT temperature of male mice measurements during room temperature environment and cold challenge at 12 weeks after injection virus. For female: control: *n* = 6, POMC-TLR4-KO: *n* = 6. Q: Representative near-infrared analysis of female mice. R: Quantification of food intake of female mice after cold exposure. For female: control: *n* = 6, POMC-TLR4-KO: *n* = 6. S: Weight and representative photographs of BAT at 14 weeks after injection in female mice. For female: control: *n* = 5, POMC-TLR4-KO: *n* = 6. T and U: Representative of H&E-stained sections and frequency distribution of adipocyte cell size of BAT and iWAT in female mice. Scale bar represents 40 μm. V: Changes in the mRNA expression levels involved in the BAT thermogenesis between two groups of mice. For female: *Ppargc1a*, *Pparγ*, and *Ucp1*, control: *n* = 6, POMC-TLR4-KO: *n* = 6. *Prdm16*, control: *n* = 6, POMC-TLR4-KO: *n* = 5. W: Western blot analysis of Ppargc1a, PPARγ, Prdm16, UCP1, and β-tubulin content in BAT of controls and POMC-TLR4-KO female mice. X: Quantification of Western blot analysis in BAT samples from controls and POMC-TLR4-KO female mice. For female: control: *n* = 5, POMC-TLR4-KO: *n* = 6. Y and Z: Immunostaining of UCP1 (Y) and quantification (Z) in BAT. Scale bar represents 20 μm. For female: control: *n* = 5, POMC-TLR4-KO: *n* = 5. Data are expressed as the mean ± SEM. ∗*P* < 0.05, ∗∗*P* < 0.01.

With no significant difference in physical activity and food intake, we assumed that TLR4 may affect the thermogenesis, which might contribute to regulate energy expenditure in mice. We found that the deletion TLR4 in the POMC neurons significantly increased the body temperature only in male mice at light phase (Fig. 3A). The body temperature in POMC-TLR4-KO male mice decreased in a time-dependent manner but still higher compared with control mice after exposure to cold environment (Fig. 3B). Infrared image analysis revealed that POMC-TLR4-KO mice exhibited elevated temperatures in the interscapular BAT regions at room temperature and exposure to cold environment (Fig. 3C, D) without an effect on food intake (Fig. 3E). In agreement with the changed body weight, energy expenditure, and interscapular surface temperature adjacent to BAT, we also found that the lower body weight of POMC-TLR4-KO male mice was consistent with the decreased weight of BAT (Fig. 3F). Meanwhile, the lower weight of BAT in POMC-TLR4-KO male mice likely resulted from the decreased size of adipocytes (Fig. 3G). The quantification of adipocyte size further confirmed the H&E staining results (Fig. 3H), which means POMC-TLR4-KO mice have less lipid accumulation. To investigate the mechanisms underlying the alteration of energy expenditure and adipocyte size in POMC-TLR4-KO mice, deletion of TLR4 in POMC neurons significantly increased mRNA levels of the thermogenic marker PPARG coactivator 1 alpha (*Ppargc1a*), peroxisome proliferator activated receptor gamma (*Pparγ*), PR domain containing 16 (*Prdm16*), and *Ucp1* in BAT (Fig. 3I). Consisting of the results observed in mRNA, the protein levels of PPARγ, Prdm16, and UCP1 in BAT also significantly increased in POMC-TLR4-KO mice (Fig. 3J, K). BAT of POMC-TLR4-KO mice showed increased UCP1 staining, which means the stimulation of BAT activity (Fig. 3L, M). However, in comparison with male mice, female POMC-TLR4-KO mice did not change the body temperature in light and dark phases (Fig. 3N, O), the BAT temperature at room temperature and cold environment (Fig. 3P, Q), and food intake in cold environment (Fig. 3R). Meanwhile, POMC-TLR4-KO female mice showed increased weight of BAT (Fig. 3S) and adipocyte size in BAT (Fig. 3T, U), which consist of increased body weight and fat mass. Deletion of TLR4 in POMC neurons did not change the mRNA levels of the thermogenic marker in BAT (Fig. 3V). Meanwhile, the protein levels of PPARγ, Prdm16, UCP1, as well as UCP1 staining in BAT also were normal between two groups of mice (Fig. 3W–Z). These results indicated that increased BAT thermogenesis might contribute to the increased energy expenditure in POMC-TLR4-KO male mice, but there are no effects in female POMC-TLR4-KO mice.

### TLR4 in POMC neurons regulates lipid homeostatis in male and female mice

**Fig. 4:**
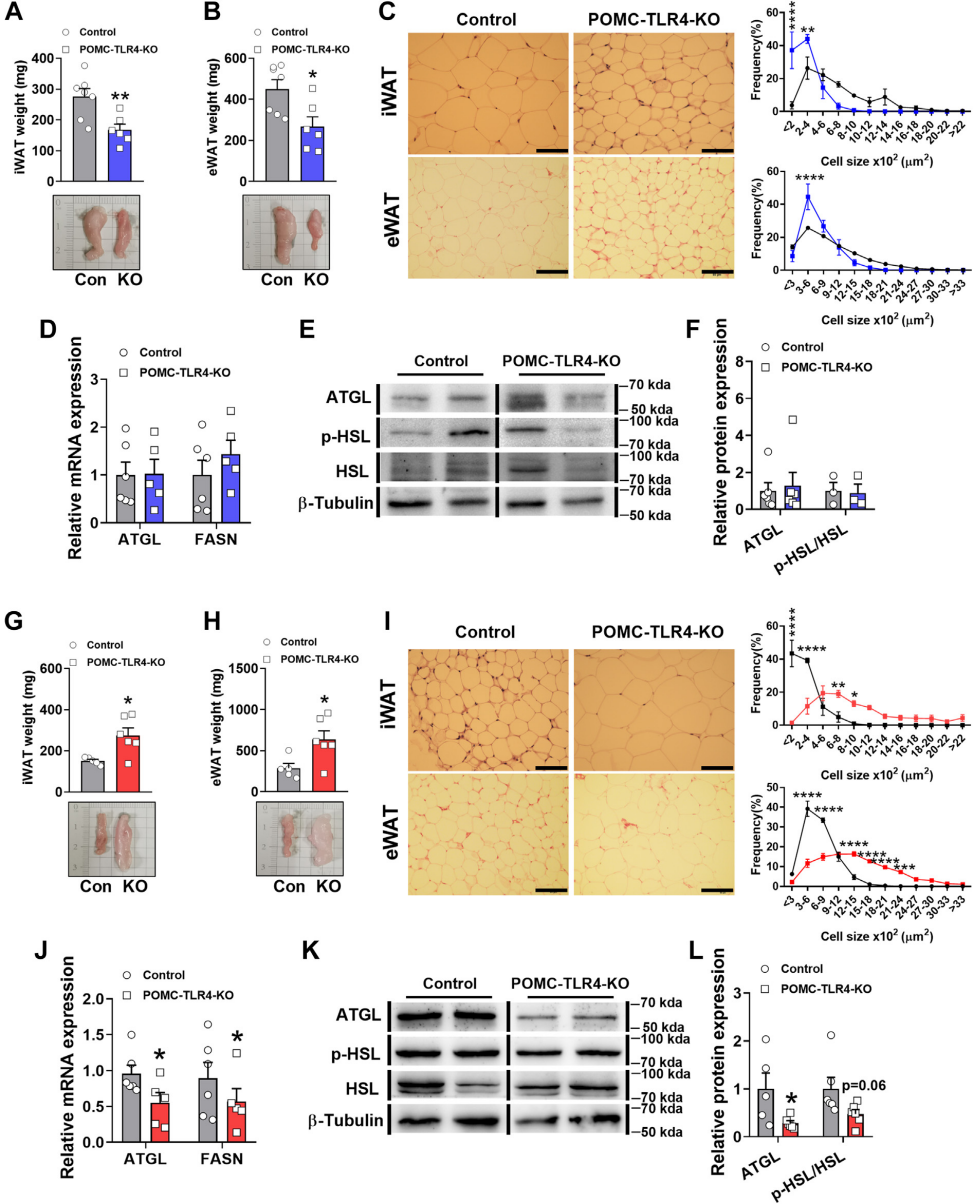
The effect of TLR4 in POMC neurons in lipid homeostasis. Male: (A, B) Weight and representative photographs of iWAT and eWAT at 14 weeks after injection in male mice. For male: control: *n* = 7, POMC-TLR4-KO: *n* = 6. C: Representative of H&E-stained sections and frequency distribution of adipocyte cell size of iWAT and eWAT in male mice. Scale bar represents 40 μm. D: Changes in the mRNA expression levels involved in iWAT lipid mobilization between the two groups of mice. For *Atgl* and *Fasn*, control: *n* = 6, POMC-TLR4-KO: *n* = 5. E: Western blot analysis of ATGL, p-HSL, and β-tubulin content in iWAT of controls and POMC-TLR4-KO male mice. F: Quantification of Western blot analysis in iWAT samples from controls and POMC-TLR4-KO male mice. For male: ATGL, control: *n* = 6, POMC-TLR4-KO: *n* = 5; p-HSL, control: *n* = 3, POMC-TLR4-KO: *n* = 3. Female: (G, H) Rectal temperature of female mice measured at room temperature environment at 12 weeks after injection. For female: control: *n* = 5, POMC-TLR4-KO: *n* = 6. I: Representative of H&E-stained sections and frequency distribution of adipocyte cell size of iWAT and eWAT in female mice. Scale bar represents 40 μm. J: Changes in the mRNA expression levels involved in iWAT lipid mobilization between the two groups of mice. For *Atgl* and *Fasn*, control: *n* = 6, POMC-TLR4-KO: *n* = 5. K: Western blot analysis of ATGL, p-HSL, and β-tubulin content in iWAT of controls and POMC-TLR4-KO female mice. L: Quantification of Western blot analysis in iWAT samples from controls and POMC-TLR4-KO female mice. For female: ATGL, control: *n* = 5, POMC-TLR4-KO: *n* = 6; p-HSL, control: *n* = 6, POMC-TLR4-KO: *n* = 6. Data are expressed as the mean ± SEM. ∗*P* < 0.05, ∗∗*P* < 0.01.

The POMC neurons are known to influence peripheral lipid metabolism and energy expenditure. BAT and iWAT are known to play critical roles in regulating lipid homeostatis. Consistent with the reduction of BAT weight in POMC-TLR4-KO mice, we also found that the male mice showed decreased weight and adipocyte size of iWAT and eWAT (Fig. 4A–C). Lipolysis is essential for thermogenesis because the fatty acids released from lipid mobilization serve as both obligatory activators for UCP1 and metabolic substrates fueling thermogenic respiration (58). However, the key components of lipid oxidation markers, such as the mRNA expression adipose triglyceride lipase (*Atgl*) and fatty acid synthase (*Fasn*) were normal in iWAT between two groups of mice (Fig. 4D). Thus, the protein expression of the ATGL and lipolytic enzyme hormone-sensitive lipase (HSL) in control and mutant iWAT was normal (Fig. 4E, F). Deletion of TLR4 in POMC neurons also increased weight and adipocyte size of iWAT and eWAT in POMC-TLR4-KO female mice (Fig. 4G–I). In addition, the mRNA levels of *Atgl* and fatty acid synthase in iWAT decreased (Fig. 4J), and protein levels of the ATGL and phosphorylated HSL (p-HSL) in iWAT also reduced significantly in female mutant mice (Fig. 4K, L).

Meanwhile, we also measured the level of triacylglycerol and NEFA in the serum. For male mice, the triacylglycerol level was not significantly different in serum, BAT, and iWAT between control and POMC-TLR4-KO male mice (supplemental Fig. S7A–C). NEFA level in serum, BAT, and iWAT also did not change (supplemental Fig. S7D–F). However, we only found that the POMC-TLR4-KO female mice exhibited significant increase of TG in iWAT (supplemental Fig. S7I) and reduction of NEFA in BAT and iWAT (supplemental Fig. S7K, L). These data suggest that TLR4 in POMC neurons can regulate lipid balance, which play an importance in regulating the body weight and energy expenditure. TLR4 in POMC neurons is a key component of the lipid mobilization in both male and female mice.

### POMC is a subpopulation of tyrosine hydroxylase-positive neurons and projects into BAT

**Fig. 5:**
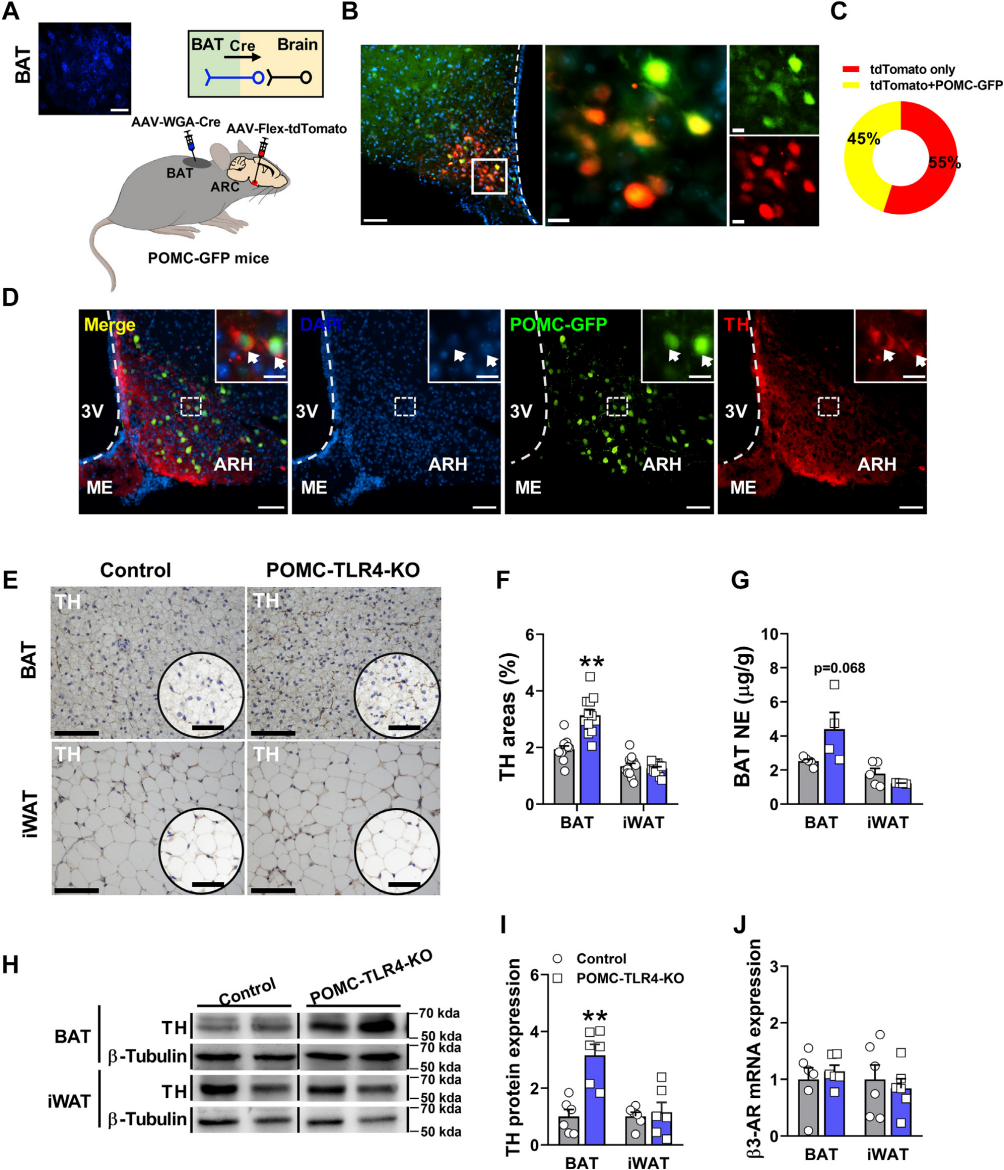
POMC is a subpopulation of TH-positive neurons and almost project into BAT. A: pAAV-CMV-WGA-NLS-Cre-P2A-EBFP2-WPRE and AAV-Flex-tdTomato injection into the BAT and ARH of 8-week-old POMC-GFP mice, respectively. Scale bar represents 200 μm. B: Immunofluorescence images showing the coexpression of POMC-GFP (green) and tdTomato (red) in ARH. Scale bar represents 200 and 40 μm. C: Percentage of tdTomato-labeled ARH neurons that are tdTomato only and tdTomato^+^ POMC^-^GFP neurons, respectively. D: A subset of POMC-GFP-positive neurons (green) coexpressed TH (red) in ARH. Scale bar represents 200 and 40 μm. E: TH staining of sympathetic innervation in the BAT and iWAT of male mice. Scale bar represents 20 and 10 μm. F: Quantification of TH staining in BAT and iWAT samples from controls and POMC-TLR4-KO male mice. For BAT: control: *n* = 12, POMC-TLR4-KO: *n* = 12; for iWAT: control: *n* = 12, POMC-TLR4-KO: *n* = 12. G: The level of NE in BAT and iWAT of controls and POMC-TLR4-KO male mice. For BAT: control: *n* = 5, POMC-TLR4-KO: *n* = 4; for iWAT: control: *n* = 5, POMC-TLR4-KO: *n* = 4. H: Western blot analysis of TH and β-tubulin content in BAT and iWAT of controls and POMC-TLR4-KO male mice. Note: β-tubulin image is same with Figure 4E because protein bands come from same batch sample. I: Quantification of Western blot analysis in BAT and iWAT samples from controls and POMC-TLR4-KO male mice. For BAT: control: *n* = 6, POMC-TLR4-KO: *n* = 6; for iWAT: control: *n* = 6, POMC-TLR4-KO: *n* = 6. J: The β3-AR mRNA expression in BAT and iWAT of controls and POMC-TLR4-KO male mice. For BAT: control: *n* = 6, POMC-TLR4-KO: *n* = 6; for iWAT: control: *n* = 6, POMC-TLR4-KO: *n* = 6. Data are expressed as the mean ± SEM. ∗*P* < 0.05, ∗∗*P* < 0.01.

POMC neurons regulate adipose tissue thermogenesis through the SNS (59). To identify if POMC neurons whether innervating or projecting to BAT, a pAAV-CMV-WGA-NLS-Cre-P2A-EBFP2-WPRE virus, which is known to undergo single and trans-synaptic retrograde transport was injected in the BAT of POMC-GFP mice. At the same time, we also injected the AAV-Flex-tdTomato (Cre dependent) into the ARH of these mice. Because the WGA-Cre can travel retrogradely, which also can induce the fluorescence of red tdTomato in the virus-injecting site of brain. tdTomato-labeled somas in the ARH were identified as the neurons that project to the BAT (Fig. 5A, B). At 4 weeks postinjection, we found the that WGA-Cre expressed blue signal in BAT. Surprisingly, there was a most 45% overlap between the POMC-GFP and tdTomato neurons (Fig. 5C). These results indicate that POMC neurons provide inputs to a portion of BAT. Next, deletion of TLR4 in POMC neurons reduced the tyrosine hydroxylase (TH) expression and POMC projected into BAT. Thus, we speculate whether POMC neuron is a part of TH-positive neurons. To address this question, we examined the TH-positive neuron expression in ARH region. Using immunofluorescence, we observed the colocalization of TH-positive neurons and GFP-labeled POMC neurons, which confirms that POMC neurons in ARH are subpopulations of TH-positive neurons (Fig. 5D).

The SNS plays a key role in the lipolytic activity and adipose tissue thermogenesis. In order to determine whether the change of BAT thermogenesis and lipid mobilization was associated with altered SNS function, we further assessed the activity of sympathetic nerve terminals and NE levels released in BAT and iWAT. First, we found the TH staining, which labels sympathetic nerve terminals, revealed a dramatic increase in sympathetic fiber density in BAT of POMC-TLR4-KO male mice compared with the control mice (Fig. 5E, F). Meanwhile, NE level in BAT was greatly increased in POMC-TLT4-KO male mice (Fig. 5G). Consistently, the protein expression of TH, an enzyme required for the synthesis of catecholamines, was significantly increased in BAT from control and POMC-TLT4-KO male mice (Fig. 5H, I). Cold stress induces the secretion of norepinephrine that activates the β3-AR in adipose tissue (32, 60). However, we did not observe the difference in the mRNA expression of β3-AR in POMC-TLR4-KO male mice (Fig. 5J). In addition, we did not find any alteration of TH staining, NE, TH protein, and β3-AR level in BAT and iWAT from control and POMC-TLT4-KO female mice (supplemental Fig. S8A–F). Together, these data suggested that POMC-specific TLR4 deficiency may stimulate thermogenesis via increasing sympathetic innervation only in BAT of male mice. Together, these results indicated that TLR4 in POMC neurons regulate the activity of sympathetic nervous system, and POMC neurons provide projection to BAT.

### Effect of deletion of TLR4 in POMC neurons on transcript expression in iWAT of TLR4^f/f^ and POMC-TLR4-KO female mice

**Fig. 6:**
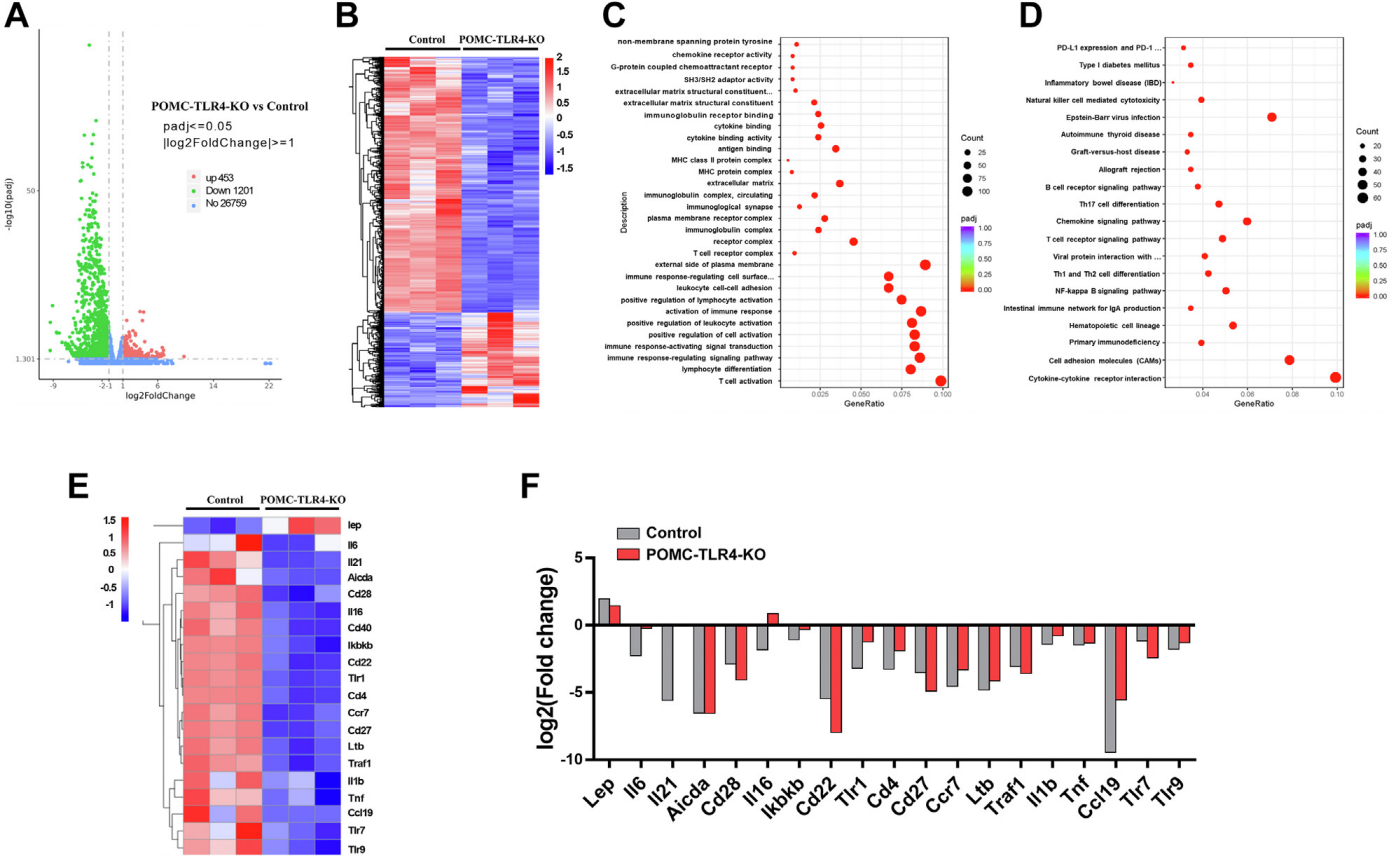
Effect of deletion of TLR4 on transcript expression in iWAT of control and POMC-TLR4-KO female mice. A: Log2 fold changes of RNA-Seq gene bodies in POMC-TLR4-KO versus control mice and the corresponding significance values displayed as log10 (*P* value). The transverse and vertical dotted lines indicate the cutoff value for differential expression. Totally, 453 and 1,201 genes were identified that had increased (red) or decreased (blue) expression levels after deleting TLR4. B: The levels of differentially expressed genes in iWAT were calculated by log2 between POMC-TLR4-KO and control mice. C: Gene Ontology (GO) enrichment analysis. D: Functional enrichment analyses using Kyoto Encyclopedia of Genes and Genomes (KEGG) pathways. E: Differentially expressed genes in immune response signaling pathway from the RNA-Seq dataset. F: Comparison of expression measured by quantitative PCR and microarray in the 19 selected genes that related with immune response.

RNA-Seq was applied to explore the potential transcriptional changes and signaling pathways in iWAT of TLR4^f/f^ and POMC-TLR4-KO female mice. RNA-Seq results revealed that there are totally 26,759 transcripts expressed in the iWAT. We found 1,654 differentially expressed genes, of which 453 were increased and 1,201 were decreased based on the 1.5-fold change (Fig. 6A, B). Gene Ontology enrichment analysis also showed that the downregulating gene waas enriched in regulation of T-cell activation, immune response-regulating signaling pathway, and activation immune response (Fig. 6C). Functional enrichment analyses using the Kyoto Encyclopedia of Genes and Genomes pathways (61, 62) revealed a significant enrichment of several major signaling pathways, including cytokine-cytokine receptor interaction, primary immunodeficiency, NF-κB signaling pathway, chemokine signaling pathway, and TLR signaling pathway (Fig. 6D). Consistently, quantitative PCR results confirmed the effects of deletion of TLR4 in POMC neurons on the expression of 19 genes involved in immune response in iWAT genes to further validate the RNA-Seq data (Fig. 6E, F). These results suggest that deletion of TLR4 altered the expression of genes involved in immune response signaling pathways.

## DISCUSSION

Our findings demonstrate that the TLR4 in POMC neurons directly modulates BAT thermogenesis and lipid homeostasis in a sex-independent manner. More specifically, thermogenesis was mediated by deletion of TLR4 in POMC neurons, which activates the SNS and ultimately leads to the increase of UCP1, BAT, temperature, and energy expenditure in male mice. On the other hand, deleting TLR4 in POMC neurons altered lipid homeostasis in iWAT, which resulted in decreasing energy expenditure and enhance body weight in female mice. Meanwhile, the decreasing function of immune system may exacerbate the development of obesity. More importantly, here we demonstrate an essential and sex-specific role of TLR4 in the POMC neurons facilitating sympathetic output in male mice and lipid balance in female mice.

We found that TLR4 in POMC neurons is an essential regulator of the whole-body energy homeostasis. In male mice, deletion of TLR4 decreased the body weight and increased the energy expenditure, including heat production, the volume of O_2_ consumption, and the volume of CO_2_ production. On the contrary, deletion of TLR4 increased the body weight and decreased the energy expenditure in female mice. But the food intake and blood glucose did not change in both male and female mice. It has been demonstrated that knockdown of TLR4 in ARH can reduce body weight and energy intake in male obese rats. However, there is no significant effect on the body weight and energy intake in chow-diet group rats. This may be because the HFD enhanced the hypothalamic inflammation, and loss-of-function mutation in TLR4 can reduce the inflammation and correct insulin and leptin resistance (35). One study has showed that losing the TLR4 adaptor myeloid differentiation primary response 88 in all brains could impair glucose and insulin tolerance (43). In our study, we only found the alteration of glucose homeostasis in male mice. From this, we cannot determine that mice have the features of glucose metabolism disorders. It has been reported that only female mice lacking TLR4 showed increased obesity but partially protected against HFD-induced obesity and insulin resistance, accompanied by less liver and fat inflammation compared with control mice. On chow diets, female TLR4^−/−^ mice showed an increase in body weight gain, fat mass, and food intake. However, there was no detectable change in energy expenditure. Meanwhile, there were no differences detected in body weight, food intake, or insulin sensitivity in male mice (37). Our previous studies have showed that the deletion of TLR4 in dopamine neurons increased the body weight and food intake in male mice (39). But TLR4 KO mice showed normal body weight gain and food intake at chow diet (63). Thus, TLR4 that is located in different neurons may play dissimilar role in the regulation of energy balance.

In our study, we also showed that TLR4 in POMC neurons modulates the thermogenesis of BAT and lipid homeostasis. More specifically, deleting TLR4 in POMC neurons stimulates BAT thermogenesis in male mice by triggering the level of some well-known molecular markers, such as Ppargc1a, PPARγ, Prdm16, and UCP1. Furthermore, the capacity of UCP1 to increase BAT activity provides an additional mechanism by which deletion of TLR4 in POMC neurons may increase energy expenditure and reduce body weight. Because of its unique role in energy expenditure, BAT has become a new target for obesity treatment and prevention. A previous study has reported that the stimulation of TLR4 by HFD or LPS is related to the decrease of core body temperature and heat production. At the same time, TLR4 deletion can prevent LPS-induced downregulation of thermogenesis (64). A recent study reported that chronic activation of pattern recognition receptors, such as NOD-like receptor or TLR inhibits the expression of BAT-specific marker genes (65). This study aligns with our finding that ablation of TLR4 enhanced the BAT-specific marker gene expression, like *Prdm16* and *Ucp1*. Moreover, activation of TLR4 in BAT induces inflammation and suppresses UCP1 expression and mitochondrial respiration (66). Interestingly, the thermogenesis of TLR4 in POMC neurons seems to be specific to male mice since the alteration of BAT activation did not occur in female mice. At the same time, deletion of TLR4 also differentially affected PPARγ and Ppargc1a expression, both of which are important for the expression of BAT markers, such as UCP1. Evidence has emerged from studies that PPARγ is related to WAT differentiation and BAT differentiation (67, 68). Ppargc1a coactivates transcription factor PPARγ bound to the promoters of target genes, such as *Ucp1*, to control gene transcription (69, 70). Our results showed that deletion of TLR4 increased both PPARγ mRNA and protein expression, whereas only enhanced Ppargc1a mRNA but not the protein expression. The differences between mRNA and protein expression of Ppargc1a may be due to the protein stability and post-transcriptionally way. The increase of thermogenic capacity in BAT likely contributes to the reduction of adiposity size and consequently body weight. However, we found that TLR4 in POMC neurons regulates lipid metabolism in adipocytes as shown in the findings of female mice. The triglyceride content of female mutants in iWAT is increased, indicating aberrant lipid metabolism. Furthermore, the expression of fatty acid mobilization markers was decreased after deleting TLR4 in POMC neurons, as represented by the lower levels of ATGL and p-HSL protein. In this sense, these changes in lipid metabolism are consistent with the finding of body mass in female mice. This result was unexpected since the deletion of TLR4 in POMC neurons leads to the different changes of BAT and iWAT in male and female mice.

Besides, given that gender could affect obesity and multiple metabolic process (71), we cannot exclude the effect of gender on the energy balance. Previous study reported that deletion of SIRT3 in POMC neurons could reduce weight gain and increased thermogenic effect in male mice but not female mice (5). Another study showed that bisphenol-A induced obesity, increased energy intake only occurred in HFD-fed female mice, whereas glucose intolerance was found in both male and female HFD-fed mice. This may be because male and female mice have unique estrogen receptor expression and their alteration after bisphenol-A exposure (72). These sex-dependent effects were also found in our study. In our results, we only find that the BAT thermogenic was increased in male mice but not female mice. Whereas the changes of lipid metabolism marker genes, like *Atgl* and *p-hsl/hsl*, occurred in female mice, which contributes to the body weight gain. However, it was reported that mRNA expression of TLR4 is elevated in obese and type 2 diabetes patients (73), and TLR4 protein is higher in males compared with females in peripheral blood mononuclear cells (74). Further research needs to be done to determine the biological mechanism by which TLR4 regulates body weight in different genders.

BAT plays an important role in the regulation of thermogenic. There is a link between CNS and peripheral fat tissue. Several studies have suggested that CNS regulates BAT function, which is a key component of adaptive thermogenesis (22, 75, 76). Besides, administration of *N*-methyl-D-aspartate or bicuculline (a γ-aminobutyric acid antagonist) to rat lateral hypothalamus (LH) increased BAT temperature. More specifically, losing alpha2delta-1 in SF1 neurons of the ventromedial hypothalamus regulates lipid metabolism (77). Specific deletion of Sh2b1 in leptin receptor neurons abrogates and disrupts BAT thermogenic function, leading to reduced core body temperature and cold intolerance (78). Ablation of SIRT3 in POMC, but not in AGRP neurons, increased BAT activity and induced browning in WAT (5). Besides, deleting SIRT1 in POMC neurons decreased SNA in WAT, thus reducing the browning of WAT (79). Deleting protein tyrosine phosphatases PTP1B (PTPN1) and tyrosine phosphatase protein (PTPN2) in POMC neurons promoted WAT browning by enhancing the SNA to adipose tissues (22). In addition, deletion of activating transcription factor 4 in POMC neurons activated the SNA in BAT, which resulted in increased UCP1 expression in BAT and NE level in serum (80). However, overexpression of SIRT6 in POMC neurons significantly decreased sympathetic nerve innervation and activity in adipose tissues, which demonstrated by decreased expression of TH and the NE levels (81). Lacking mesencephalic astrocyte-derived neurotrophic factor in POMC neurons ultimately decreased SNA and thermogenesis in BAT (82).

Chemogenetically activated Vglut2 neurons in LH increased the BAT temperature. However, using the PRV trans-synaptic, retrograde tracing virus-labeled neurons functionally connected to BAT in the brain, including the including S1HL (primary sensory cortex, hindlimb region), paraventricular hypothalamus, LH, ventromedial hypothalamus , periaqueductal gray, and raphe pallidus nucleus (83). Meanwhile, the POMC neurons also connect to the BAT and iWAT (84). However, it is unclear if there is a direct link between POMC neurons and BAT because of the PRV retrograde multisynaptic tracing. Here, we report that POMC neurons do have collateral projections to BAT and iWAT directly using WGA virus. These results imply that thermogenesis regulation may be achieved by affecting the activity and function of POMC neurons that connect with BAT.

It is worth mentioning that we showed that levels of TH and NE in BAT are increased, which might, at least in part, drive the elevation sympathetic nervous system activity and sympathetic tone in male POMC-TLR4-KO mice. The SNS regulates thermogenesis in BAT and lipolysis in WAT (85). Sympathetic innervation system of adipose tissue promotes lipolysis and thermogenesis through NE signaling (86). However, we didnot detect the significant difference of β3-AR expression in BAT between control and POMC-TLR4-KO male mice.

β3-AR activation stimulates lipolysis through the mobilization of lipids from the WAT and increases thermogenesis in BAT (87). Deletion of UCP1 in BAT was reported to be unresponsive to NE, indicating that importance of UCP1 in β3-AR signaling mediated thermogenesis (88). TH is the rate-limiting enzyme in the biosynthesis of the catecholamines, like DA and NE. Besides, TH neurons are well known to project to brainstem and spinal autonomic regulatory centers and integrate sympathetic outflow (89). ARH NPY signaling reduced TH expression in the paraventricular nucleus (PVN), which controls BAT thermogenesis and sympathetic output via TH neurons in PVN (90). Recent study showed that the reduction of NPY in ARH is accompanied by the increase of UCP1 expression in BAT, resulting in the activation of thermogenesis, suggesting that NPY in ARH negatively regulates BAT function (91). We examined the distribution of TH-expressing neurons in the ARH to determine if POMC neuron is a separate population of TH-positive neurons. Consistently, we observed that a small portion of POMC neurons coexpress TH, which means POMC neurons in ARH are a subset of TH-positive neurons. Our previous study has reported that deletion of TLR4 in dopamine neurons reduced the DA activity and TH protein expression in VTA. However, future studies are essential to further dissect out the POMC neuronal activity to the regulation of TH expression in periphery.

Using RNA-Seq, we found that the function of immune system was suppressed in iWAT of female POMC-TLR4-KO mice, including several immune response-regulating pathways. Recent studies have reported that obesity is related to immune dysfunction by observing that obese subjects have a higher rate of infections and impaired wound healing. Excess body fat is accompanied by lowing T- and B-cell mitogen-induced proliferation (92). Meanwhile, other research has proved that animals with obesity have impaired the immune response (93, 94). Obese strains have a lower phagocyte activity by macrophages and a lower expression of proinflammatory-related cytokines (95). Meanwhile, the obese strains had lower activity of macrophages and expression of proinflammatory cytokines, which is consistent with our results. In individuals, Gottschlich *et al.* (96)also showed that infectious diseases were higher in obese patients compared with lean men. In addition, obese patients have the lower production of antibodies after damaging vaccination (97). Chandra and Au (94) also found that 38% of obese individuals showed impairment of cell-mediated immune responses, such as lower T- and B-cell mitogen-induced lymphocyte proliferation and reduced ability of polymorphonuclear leukocytes to kill intracellular bacteria. In addition, obesity appears to reduce lymphocyte immune function and NK cell activity in people over 60 years of age. From this point, it is clear that the development of obesity damages the immune function. However, it is unknown that if the dysfunction of immune is a major contributor to obesity and the alteration of immune underlies the onset of obesity. Further research still needs to illustrate the relationship and feedback between altered immune response in obesity.

Although our study first reveals deleting TLR4 in POMC neurons increases UCP1 expression, which contributes to the thermogenesis of BAT and energy expenditure in male mice but not in females. However, the mechanism of how TLR4 in POMC neurons in females regulates body weight and metabolism remain to be depicted. Indeed, it is possible that TLR4 levels in other hypothalamic regions will compensatory changes because we used the viral strategy to delete TLR4 in POMC neurons. For example, the mRNA of TLR4 is a little higher (no difference) in PVN and VTA both in male and female POMC-TLR4-KO mice compared with control group, which does not fully allow us to study the specific role of TLR4 in POMC neurons. In the future, more specific target to TLR4 in POMC neurons need to be addressed and studied.

In summary, our result supports that TLR4 in POMC neurons is a key regulator of thermogenesis and lipid balance in a sex-dependent manner. Deleting TLR4 in POMC neurons increases UCP1 expression, which contributes to the thermogenesis of BAT and energy expenditure in male mice. However, deleting TLR4 in POMC neurons decreases the lipid lipolysis and immune response, which may decrease energy expenditure and increase the body weight in female mice.

## Data availability

The original data presented during this study are available from the corresponding authors upon reasonable request.

## Supplemental data

This article contains supplemental data.

## Conflict of interest

The authors declare that they have no conflicts of interest with the contents of this article.
