# Supplementary material for: TLR4 in POMC neurons regulates thermogenesis in a sex-dependent manner
Source: J Lipid Res. 2023 Apr 6;64(5):100368. doi: 10.1016/j.jlr.2023.100368 (PMC10205441; doi:10.1016/j.jlr.2023.100368)
Supplement: Supporting information [file mmc1.docx]

**TLR4 in POMC neurons regulates thermogenesis in a sex dependent manner**

**Supplementary materials**

**Identifying the gene type of mice**

Genotyping was conducted on tail biopsies harvested at weaning via polymerase chain reaction and gel electrophoresis. For verification of the TLR4^flox/flox^ alleles, the PCR products were amplified using the primers 5′-TGA CCA CCC ATA TTG CCT ATA C-3′ and 5′-TGA TGG TGT GAG CAG GAG AG-3′. The POMC-GFP allele was verified using the primers 5′-AAG TTC ATC TGC ACC ACC G-3′ and 5′-TCC TTG AAG AAG ATG GTG CG-3′.

**Body weight and composition**

The body weight of control and POMC-TLR4-KO mice was monitored after surgery every week. Body composition of mice was analyzed by quantitative nuclear resonance at 12 weeks after surgery. (QMR, Niumag Corporation, Shanghai, China).

**Glucose and insulin tolerance tests**

The mice were weighed, and blood glucose were measured at time 0 for baseline glucose level. For the glucose tolerance test (GTT), mice aged 20 weeks were fasted overnight, followed by intraperitoneal glucose injection (1 g/kg body weight); For the insulin tolerance test (ITT), mice aged 20 weeks were fasted for 4 hours during the light phase, followed by intraperitoneal insulin injection (0.75 U/kg body weight).

**Metabolic analysis**

To measure metabolic rate, the 24 weeks mice were housed individually in a combined indirect calorimetry system (Promethion Metabolic Screening Systems, Sable systems International, North Las Vegas, NV, USA). The systems consist of a combination of feeding sensors for automated online measurement. The calorimetry system is an open-circuit system that determines heat generation, O_2_ consumption, CO_2_ production, and the respiratory exchange ratio (RER). Data were collected after 24 h of adaptation in acclimated, individually housed mice. Food intake data were further used for meal microstructure analysis.

**Elevated plus-maze test**

The test maze was made of metal with two open arms (33 × 5 cm) and two closed arms (33 × 5 × 15 cm) and was elevated 60 cm above the floor. Mice were placed in the center of the maze facing the open arm and allowed to explore the maze freely for 5 min. The movement was recorded for 30 min using a video camera fixed overhead. The open arm entries and the time spent in the open arms were analyzed using the software (SuperMaze, Xinruan Corporation, Shanghai, China). After each session, the maze was cleaned with 75% alcohol solution.

**Open-field test**

The open-field test was carried out in a clear metal open-field area (50 × 50 × 30 cm). Individual mice were placed in the center area of the chamber, and the paths of animals were recorded by a video camera. Mice were first placed into the center of the area and allowed to explore for 5 min. The time spent in the center field and entries into the center field were analyzed using the software (SuperMaze, Xinruan Corporation, Shanghai, China). After each session, the maze was cleaned with 75% alcohol solution.

**Serum and tissue collection and measurements**

Serum of mutants and controls mice was collected after fasting 24 hours. In the end, mice were euthanized via cervical dislocation, and blood samples were collected via retro-orbital bleeding. Meantime, BAT and iWAT were dissected and frozen immediately in liquid nitrogen. Serum insulin was measured using ELISA kit (RF8164, Ruifan Biological Technology Co., Ltd., Shanghai, China). Serum triglyceride, free thyroid hormone 3 and thyroid hormone 4 were measured by ELISA kit (H350-1, H224 and H225 Nanjing Jiancheng Biological Engineering ReseArch Institute, Jiangsu, China). Serum and tissues NEFAs and NE were determined by the ELISA kits (RF7916 and RF 8448, Ruifan Biological Technology Co., Ltd., Shanghai, China). Serum estrogen was tested by the ELISA kits (CSB-E07280m, CUSABIO, Wuhan, China)

**RNA extraction and Real time RT PCR**

Control and POMC-TLR4-KO mice of both sexes were sacrificed via cervical dislocation, and the areas of brain (ARH, PVN, VTA and DRN), BAT and iWAT samples were immediately dissected on ice and frozen in liquid nitrogen after dissection and stored at −80 °C until further use. Total RNA was extracted with the Hipure Universal RNA Kit (Magen Biotech Co., Ltd. Guangzhou, China). The cDNA was generated using the Color Reverse Transcription Kit (A0010CG, EZBioscience, USA). Gene quantification was performed by mixing cDNA, the SYBR reagent (A0012-R2, EZBioscience, USA) and the primers (Sangon, Guangzhou, China). The primer sequences used for reverse transcription PCR and quantitative real-time PCR can be found in the Supplementary Table 1. The processed for real-time PCR quantification using the QuantStudio^TM^ 3 Real-Time PCR system (ThermoFisher, Waltham, MA, USA).

**Western blot assay**

Total protein was extracted from BAT and iWAT samples using RIPA lysis buffer that contained 1 mM phenylmethylsulfonyl fluoride (PMSF). Protein concentration was determined using a BCA protein assay kit (Thermo Fisher Scientific). Protein samples were separated by 10% SDS-PAGE and transferred to a PVDF membrane and then blocked with 6% (wt/vol) nonfat dry milk in TBST at room temperature. Membranes were then subjected to immunoblotting with the primary antibody against TLR4 (1:2000, sc-293072, Santa Cruz, USA), TH (1:1000; MAB318, Millipore, USA), Ppargc1a (1:2000, NBP1-04676SS, Novus, USA), PPAR γ (1:2000, 81B8, CST, USA), Prdm16 (1:1000, bsm-51634M, Bioss, China), UCP1 (1:2000, D906X, CST, USA), ATGL (1:1000, A5126, Abclonal, China), p-HSL (1:1000, AP1151, Abclonal, China), HSL (1:1000, A15689, Abclonal, China), and β-Tubulin (BS 1482M, Bioworld, USA) at 4°C overnight, followed by goat-anti-rabbit or mouse horseradish peroxidase (HRP)-conjugated secondary antibody (1:50000, BS13278 or BS12478, Bioworld, USA) for 1.5 h at room temperature. Protein expression was measured using a Fluorescence Imaging System (Tanon-5200, Shanghai, China) and normalized.

**Histomorphology of adipose tissues and immunohistochemistry for UCP-1 and TH**

BAT and iWAT and were fixed for 48 h in 10% formalin buffer, and then were dehydrated and embedded in paraffin by a standard procedure cut into 4 mm sections. For H&E staining, sections were stained with hematoxylin and eosin (H&E) according to the manufacturer’s instructions. The fat cell size of BAT and iWAT were measured in H&E-stained sections of three individual samples in each group. The average diameter (μm) of adipocyte were analyzed using Image-J software (National Institutes of Health, NIH). The detection of UCP-1 and TH in BAT was performed using anti-UCP1 (1:500; GB112174, Servicebio, China), anti-TH (1:500; GB11181, Servicebio, China) by Wuhan Servicebio Technology Co., Ltd. China. Images were observed, and positive areas were counted by using Image-J software. Three mice per experimental group were used.

**Statistical analysis**

All data are presented as the means ± standard error of the mean (SEM). Statistical analysis was performed using GraphPad Prism 8.0. Student’s t test was used to determine significant differences between the control and POMC-TLR4-KO groups. Two-way ANOVA was used for multiple comparisons followed by post hoc Bonferroni test. A significance (alpha) level of *P* < 0.05 was considered statistically significant.

**Supplementary results**


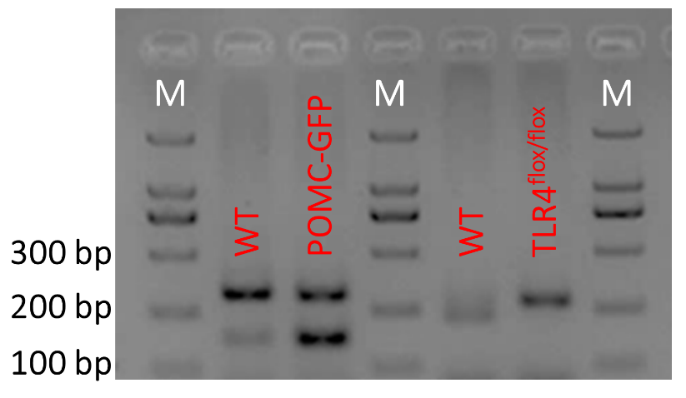


**Fig. S1:** POMC-GFP and TLR4 homozygote genotyping was conducted via polymerase chain reaction and gel electrophoresis.


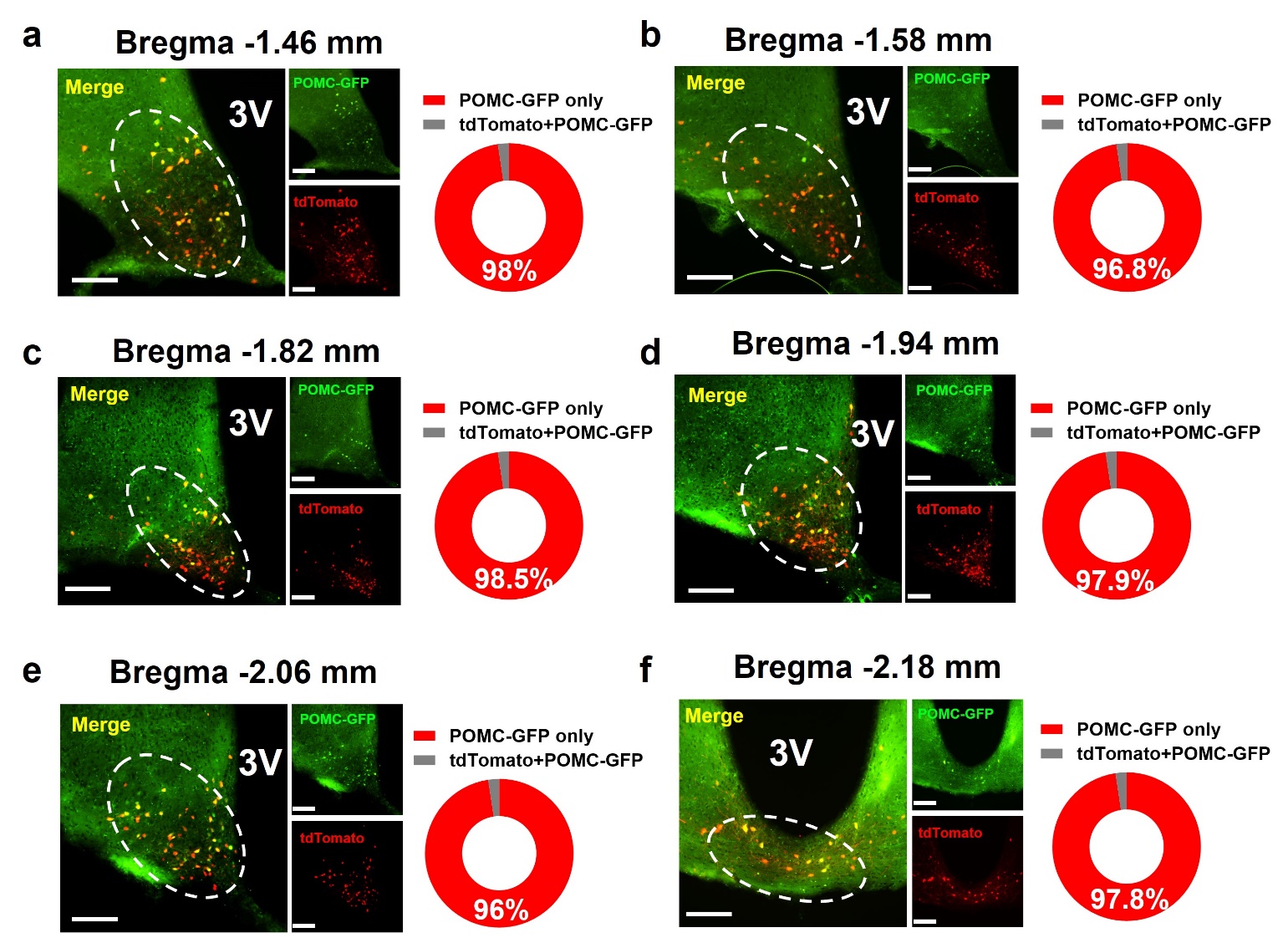


**Fig. S2:** (a-f) Fluorescence immunohistochemistry for colocalization of POMC-GFP and tdTomato-labeled Cre-dependent GFP neurons in the six bregma coordinates (bregma -1.46 mm, -1.58 mm, -1.82 mm, -1.94 mm, -2.06 mm and -2.18 mm) corresponding to anterior, medial, and posterior ARH. Scale bars: 200 μm.


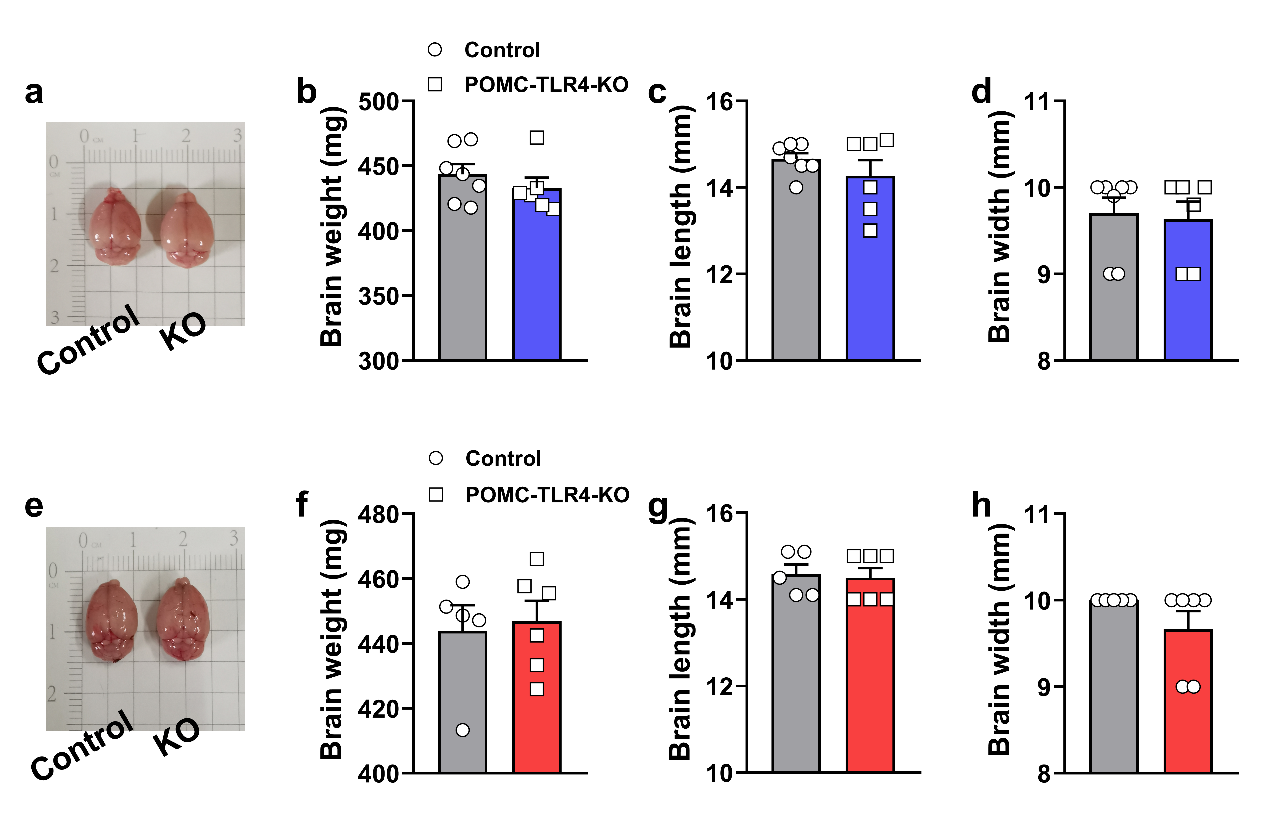


**Fig. S3:** Deletion of TLR4 in POMC neurons did not affect the brain weight and size.

(a) Representative figures showing the brain length and width of control and POMC-TLR4-KO male mice. (b) Brain weight. Control: n=7, POMC-TLR4-KO: n=6. (c) Brain length. Control: n=7, POMC-TLR4-KO: n=6. (d) Brain width. Control: n=7, POMC-TLR4-KO: n=6. (d) Representative figures showing the brain length and width of control and POMC-TLR4-KO female mice. (b) Brain weight. Control: n=5, POMC-TLR4-KO: n=6. (c) Brain length. Control: n=5, POMC-TLR4-KO: n=6. (d) Brain width. Control: n=5, POMC-TLR4-KO: n=6. Data are expressed as the mean ± SEM. **P* < 0.05, ***P* < 0.01.


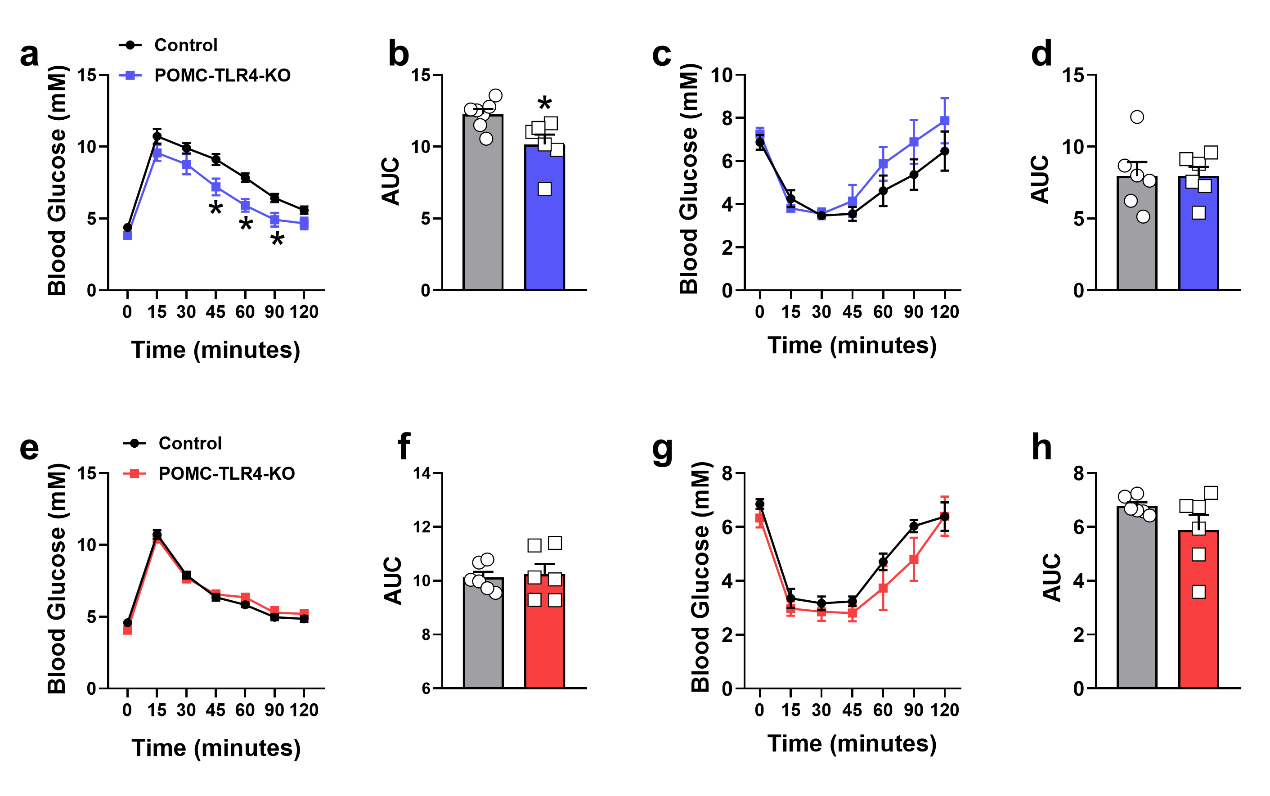


**Fig. S4:** Effect of TLR4 in POMC neuron on glucose metabolism and insulin sensitivity.

(a-d) Graphs showing the results of glucose tolerance test (a and b) and insulin tolerance test (c and d) in 21 weeks old male and female control and POMC-TLR4-KO mice. For male: Control: n=6, POMC-TLR4-KO: n=6; For female: Control: n=6, POMC-TLR4-KO: n=6. Data are expressed as the mean ± SEM. **P* < 0.05, ***P* < 0.01.


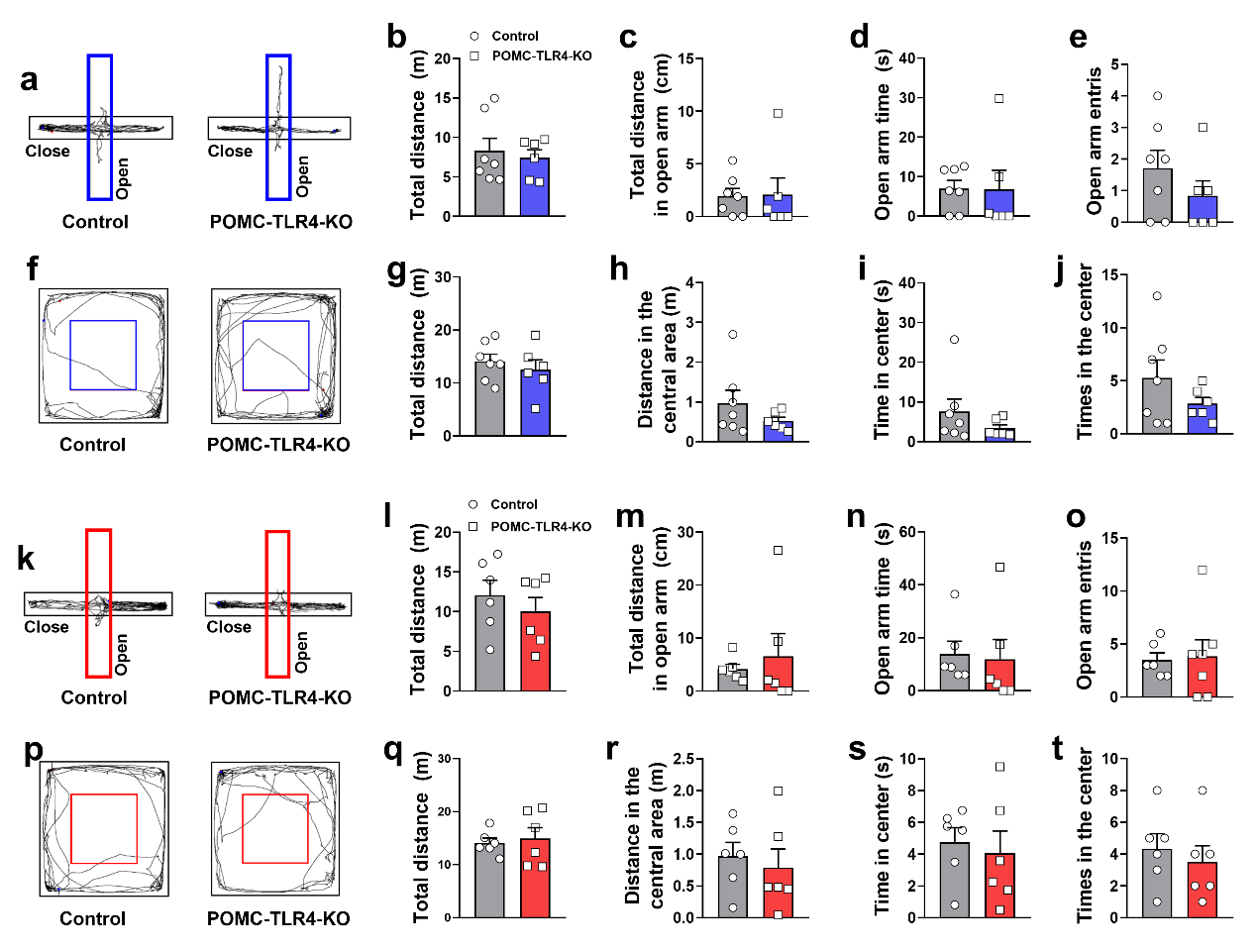


**Fig. S5:** Ablation of TLR4 in POMC neurons did not affect the anxiety related behavior.

**Male:** (a) Representative trace plot of control and POMC-TLR4-KO mice in EPM test. (b) Total travel distance; TLR4^f/f^: n=7, POMC-TLR4-KO: n=6. (c) Total travel distance in open arm; TLR4^f/f^: n=7, POMC-TLR4-KO: n=6. (d) open arm time; TLR4^f/f^: n=7, POMC-TLR4-KO: n=6. (e) open arm entries; TLR4^f/f^: n=7, POMC-TLR4-KO: n=6. (f) Representative trace plot of control and POMC-TLR4-KO mice in OFT test. (g) Total travel distance; TLR4^f/f^: n=7, POMC-TLR4-KO: n=6. (h) Travel distance in central area; TLR4^f/f^: n=7, POMC-TLR4-KO: n=6. (i) Time in center; TLR4^f/f^: n=7, POMC-TLR4-KO: n=6. (j) Times in the center; TLR4^f/f^: n=7, POMC-TLR4-KO: n=6. **Female:** (k) Representative trace plot of control and POMC-TLR4-KO mice in EPM test. (l) Total travel distance; TLR4^f/f^: n=6, POMC-TLR4-KO: n=6. (m) Total travel distance in open arm; TLR4^f/f^: n=6, POMC-TLR4-KO: n=6. (n) open arm time; TLR4^f/f^: n=6, POMC-TLR4-KO: n=6. (o) open arm entries; TLR4^f/f^: n=6, POMC-TLR4-KO: n=6. (p) Representative trace plot of control and POMC-TLR4-KO mice in OFT test. (q) Total travel distance; TLR4^f/f^: n=6, POMC-TLR4-KO: n=6. (r) Travel distance in central area; TLR4^f/f^: n=6, POMC-TLR4-KO: n=6. (s) Time in center; TLR4^f/f^: n=6, POMC-TLR4-KO: n=6. (t) Times in the center; TLR4^f/f^: n=6, POMC-TLR4-KO: n=6. Data are expressed as the mean ± SEM. **P* < 0.05, ***P* < 0.01.


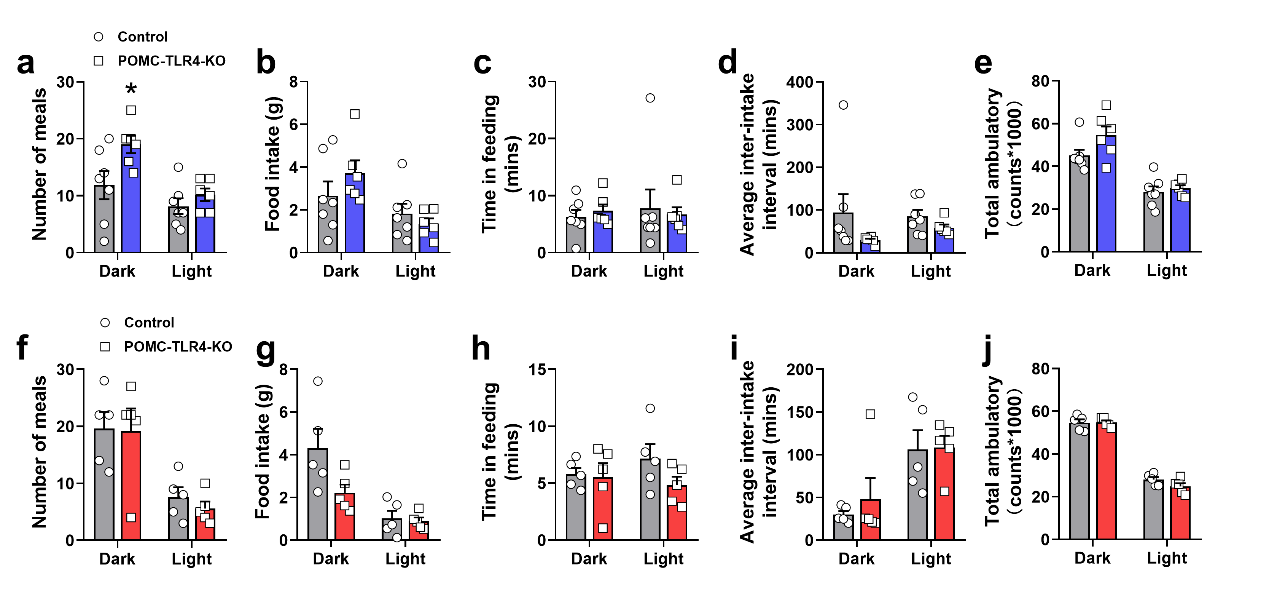


**Fig. S6:** The effects of TLR4 in POMC neurons in multiple feeding behavior and locomotor activity of mice from metabolic cages.

**Male:** (a) Number of meals; Control: n=7, POMC-TLR4-KO: n=6. (b) Food intake; Control: n=7, POMC-TLR4-KO: n=6. (c) Time spent in feeding; Control: n=7, POMC-TLR4-KO: n=6. (d) Average inter-intake interval; Control: n=7, POMC-TLR4-KO: n=6. (e) Locomotor activity; Control: n=7, POMC-TLR4-KO: n=6. **Female:** (f) Number of meals; Control: n=5, POMC-TLR4-KO: n=5. (g) Food intake; Control: n=5, POMC-TLR4-KO: n=5. (h) Time spent in feeding; Control: n=5, POMC-TLR4-KO: n=5. (i) Average inter-intake interval; Control: n=5, POMC-TLR4-KO: n=5. (j) Locomotor activity; Control: n=5, POMC-TLR4-KO: n=5. Data are expressed as the mean ± SEM. **P* < 0.05, ***P* < 0.01.


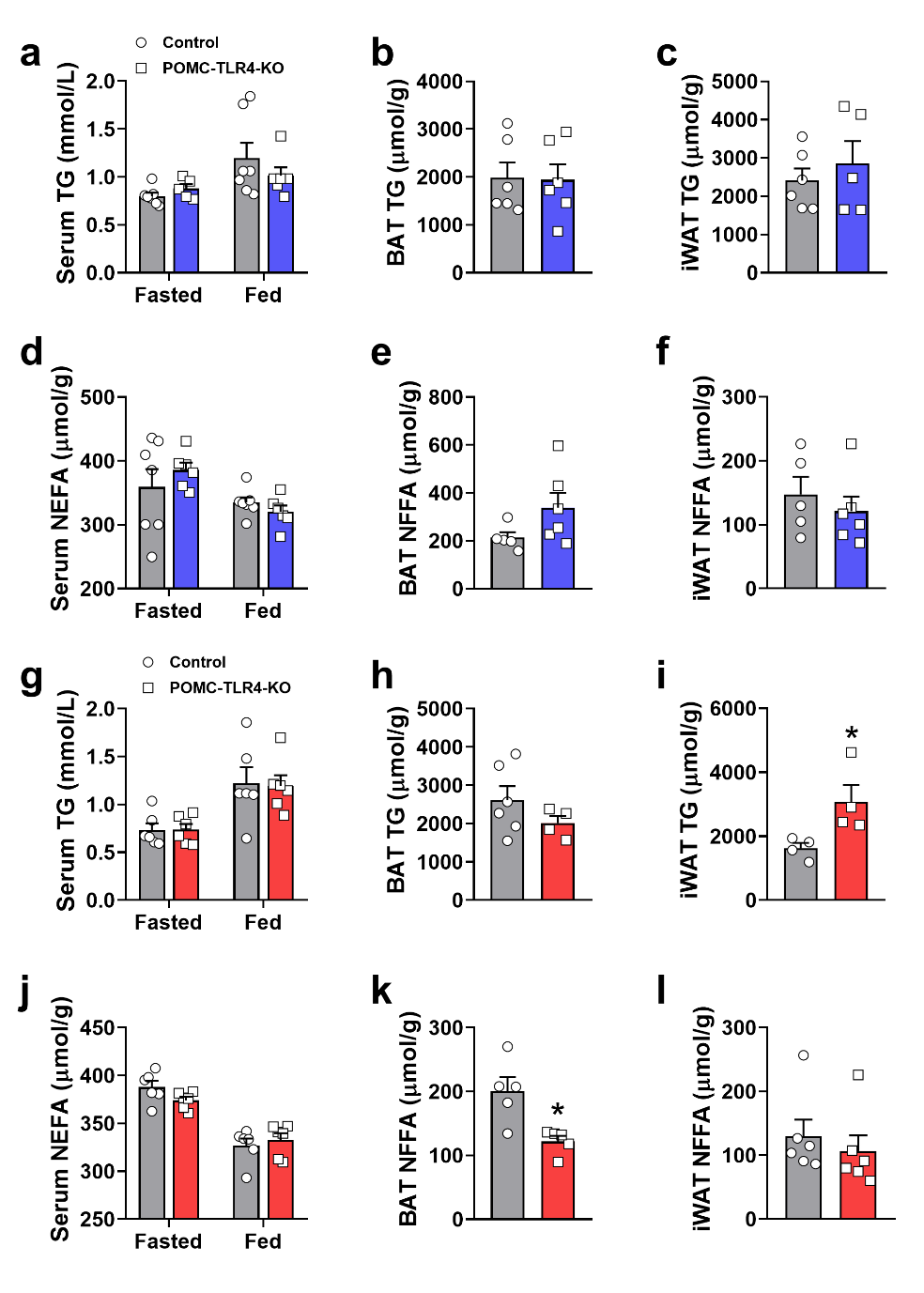


**Fig. S7.** POMC-TLR4-KO female mice have alterations in lipid balance.

**Male:** (a) Level of TG in serum under fasted and fed condition. For male: control: n=7, POMC-TLR4-KO: n=6. (b) Level of TG in BAT. For male: control: n=6, POMC-TLR4-KO: n=6. (c) Level of TG in iWAT. For male: control: n=6, POMC-TLR4-KO: n=5. (d) Level of NEFA in serum under fasted and fed condition. For male: control: n=7, POMC-TLR4-KO: n=6. (e) Level of NEFA in BAT. For male: control: n=5, POMC-TLR4-KO: n=6. (f) Level of NEFA in iWAT. For male: control: n=5, POMC-TLR4-KO: n=6. **Female:** (g) Level of TG in serum under fasted and fed condition. For female: control: n=6, POMC-TLR4-KO: n=6. (h) Level of TG in BAT. For female: control: n=6, POMC-TLR4-KO: n=4. (i) Level of TG in iWAT. For female: control: n=4, POMC-TLR4-KO: n=4. (j) Level of NEFA in serum under fasted and fed condition. For female: control: n=6, POMC-TLR4-KO: n=6. (k) Level of NEFA in BAT. For female: control: n=5, POMC-TLR4-KO: n=5. (l) Level of NEFA in iWAT. For female: control: n=6, POMC-TLR4-KO: n=6. Data are expressed as the mean ± SEM. **P* < 0.05, ***P* < 0.01.


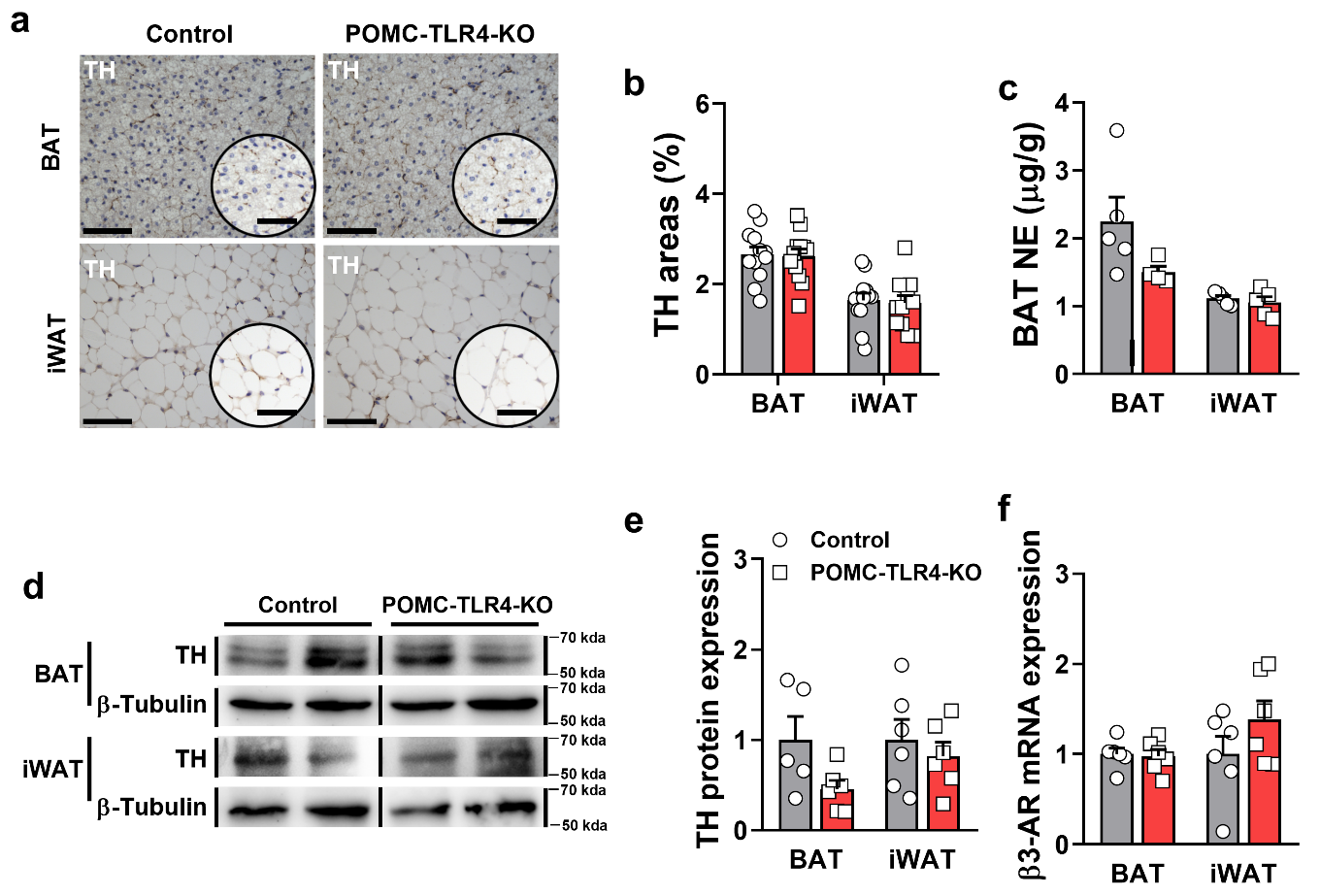


**Fig. S8.** Deletion TLR4 in neurons did not affect sympathetic outflow in female mice.

(a) TH staining of sympathetic innervation in the BAT and iWAT. Scale bar, 20 μm and 10 μm. (b) Quantification of TH staining in BAT and iWAT samples from controls and POMC-TLR4-KO female mice. For BAT: control: n=12, POMC-TLR4-KO: n=12; For iWAT: control: n=12, POMC-TLR4-KO: n=12. (c) The level of NE in BAT and iWAT of controls and POMC-TLR4-KO male mice. For BAT: control: n=5, POMC-TLR4-KO: n=4; For iWAT: control: n=5, POMC-TLR4-KO: n=5. (d) Western blot analysis of TH and β-Tubulin content in BAT and iWAT of controls and POMC-TLR4-KO female mice. Note: β-Tubulin image is same with figure 3w and 4k because protein bands come from same samples. (e) Quantification of western blot analysis in BAT and iWAT samples from controls and POMC-TLR4-KO male mice. For BAT: control: n=5, POMC-TLR4-KO: n=6; For iWAT: control: n=6, POMC-TLR4-KO: n=6. (f) The β3-AR mRNA expression in BAT and iWAT of controls and POMC-TLR4-KO male mice. For BAT: control: n=6, POMC-TLR4-KO: n=6; For iWAT: control: n=6, POMC-TLR4-KO: n=6. Data are expressed as the mean ± SEM. **P* < 0.05, ***P* < 0.01.
